# Improved Biocompatible, Flexible Mesh Composites for Implant Applications via Hydroxyapatite Coating with Potential for 3-Dimensional Extracellular Matrix Network and Bone Regeneration

**DOI:** 10.1021/acsami.1c09034

**Published:** 2021-06-07

**Authors:** Armaghan Naderi, Bin Zhang, Jorge A. Belgodere, Kaushik Sunder, Genevieve Palardy

**Affiliations:** † Department of Mechanical and Industrial Engineering, Louisiana State University, Baton Rouge, Louisiana 70803, United States; ‡ Department of Biological & Agricultural Engineering, Louisiana State University and Agricultural Center, Baton Rouge, Louisiana 70803, United States

**Keywords:** hydroxyapatite, metallic mesh substrate, cranioplasty, coating, sol−gel

## Abstract

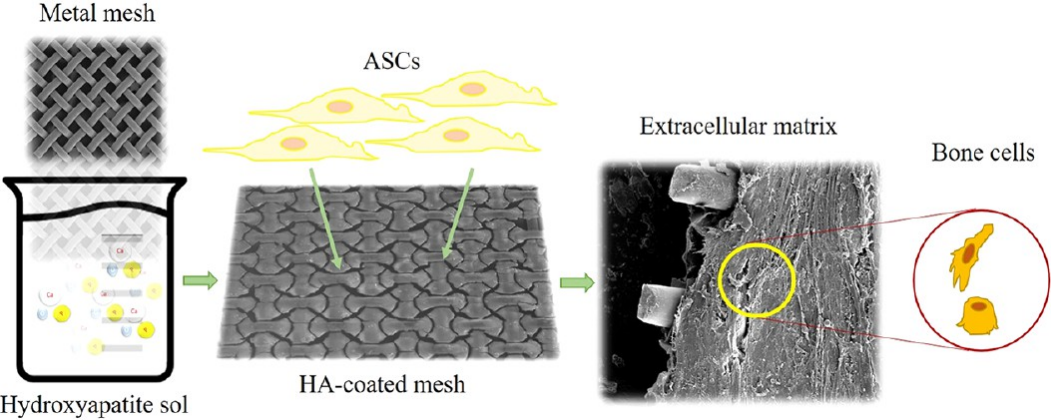

Hydroxyapatite (HA)-coated metals are biocompatible composites, which have potential for various applications for bone replacement and regeneration in the human body. In this study, we proposed the design of biocompatible, flexible composite implants by using a metal mesh as substrate and HA coating as bone regenerative stimulant derived from a simple sol–gel method. Experiments were performed to understand the effect of coating method (dip-coating and drop casting), substrate material (titanium and stainless steel) and substrate mesh characteristics (mesh size, weave pattern) on implant’s performance. HA-coated samples were characterized by X-ray diffractometer, transmission electron microscope, field-emission scanning electron microscope, nanoindenter, polarization and electrochemical impedance spectroscopy, and biocompatibility test. Pure or biphasic nanorod HA coating was obtained on mesh substrates with thicknesses varying from 4.0 to 7.9 μm. Different coating procedures and number of layers did not affect crystal structure, shape, or most intense plane reflections of the HA coating. Moduli of elasticity below 18.5 GPa were reported for HA-coated samples, falling within the range of natural skull bone. Coated samples led to at least 90% cell viability and up to 99.5% extracellular matrix coverage into a 3-dimensional network (16.4% to 76.5% higher than bare substrates). Fluorescent imaging showed no antagonistic effect of the coatings on osteogenic differentiation. Finer mesh size enhanced coating coverage and adhesion, but a low number of HA layers was preferable to maintain open mesh areas promoting extracellular matrix formation. Finally, electrochemical behavior studies revealed that, although corrosion protection for HA-coated samples was generally higher than bare samples, galvanic corrosion occurred on some samples. Overall, the results indicated that while HA-coated titanium grade 1 showed the best performance as a potential implant, HA-coated stainless steel 316 with the finest mesh size constitutes an adequate, lower cost alternative.

## 1 Introduction

Cranioplasty is a type of surgery carried out to reconstruct or replace skull defects, following traumatic brain injury (TBI), extraction of cranial tumors, skull malformations, or ischemic or hemorrhagic strokes.^1,2^ The result of the cranioplasty surgery depends on many factors, such as surgical skills and repair method, fit of contiguous soft tissues, as well as size and location of the skull defect.^3,4^ Free vascularized bone grafts, osteoinductive growth factors, and medical biomaterials are the most common repair methodologies developed in the past few years.^5^ Autograft and allograft bone donor shortage, complexity of reshaping the harvested bone, bone graft resorption, and risks of harvesting bone grafts are limitations to using bone grafts as an implant in cranioplasty.^5^ Osteoinductive growth factors can stimulate other parts of a patient’s body, including cartilage, such as hip and elbow joints, to form a new bone. However, the new bone can tear through the muscles in extreme cases and lead to heterotopic ossification (HO) in other joints.^6^

Biomaterials for cranioplasty are promising as they can reconstruct cranial defects, while presenting advantages with regards to biocompatibility, nontoxicity and aesthetics without major side effects.^7^ There are generally three categories of biomaterials used for cranioplasty: polymers (including polymer matrix composites), metals, and bioceramics. Polymers or polymer composites, such as methyl methacrylate (MMA), porous polyethylene (PE), and customized polyetheretherketone (PEEK) for large skull defects, have complications due to potential inflammation, infection, implant exposure, skin penetration, and radiolucency.^5,8−10^ Another problem is that custom-made implants are time-consuming and costly to design and manufacture. They need to be designed according to computed tomography (CT) scans or other 3D images and, then, manufactured by molding, die casting, or 3D printing.^2,11,12^ All these processes require several months to be carried out by authorized suppliers. Metallic biomaterials, generally stainless steel and coated cobalt–chromium alloys, were popular as load-bearing implants in the past (1938–1960s), but possessed poor corrosion properties.^13^ In recent years, titanium and titanium alloy (Ti–6Al–4V) have gained popularity. They are mostly used as bulk or plate implants for hip and joints, and custom-made or mesh-like implants for cranioplasty. However, the lack of isoelasticity of skeleton and bone, as well as cytotoxicity from the release of ions (e.g., Ti^4+^, aluminum (Al), and vanadium(V)), are potentially harmful to the human body’s immune system. Additionally, all metallic implants restrict the use of magnetic resonance imaging (MRI) and cone beam X-ray imaging for medical diagnosis.^5,8,14^

Bioceramics are another type of promising materials for cranioplasty. For instance, tricalcium phosphate (TCP) and hydroxyapatite (HA, Ca_10_(PO_4_)_6_(OH)_2_) can be used as bulk, scaffold, powder, cement, filling for bone fractures, and coating on bioinert materials.^8,15−17^ Bioceramics naturally possess good osteoinduction and osteoconduction. Several histological studies in the literature presented formation of bone in-growth on different forms of porous and nonporous HA implants and calcium phosphate scaffolds.^5,16−18^ However, their low strength and brittleness limit their application to non-load-bearing human body skeleton, such as cranial and mandible bone.^8,15^ Combining bioceramics and metallic substrates by using HA coatings has shown promising advantages, such as an increase of implant strength.^8,19,20^ HA coating is expected to improve biocompatibility, while displaying optimal porosity, good adhesion to the substrate, high crystallinity, and proper stoichiometry (Ca/P ratio of 1.67).^20,21^

There are several methods to deposit HA coating on a substrate: electrophoretic deposition, electrochemical deposition, pulsed laser deposition, hot isostatic pressing, plasma spraying, biomimetic coating, sputter coating, spin coating, and dip-coating sol–gel.^20,21^ The dip-coating sol–gel process is an inexpensive, low processing temperature method leading to good adhesion and high surface uniformity for complex shape substrates, with thin coating thickness from hundreds of nanometers to a few millimeters.^19,20,22^ Both dip-coating and drop casting methods can be used from HA sol–gel for most substrates with different shapes to prepare a combined implant for cranioplasty operated at decompressive craniectomy in emergency operations. The implant should be flexible to allow for customizability in an emergency, easy to cut and use, biocompatible with osteointegration properties, and nontoxic.

In this study, we hypothesize that a thin HA layer coated on both sides of a metallic mesh substrate can improve bone regeneration by forming a 3-dimensional (3D), through-the-thickness extracellular matrix (ECM) network, while protecting the implant from losing ions over time. Therefore, this research aims to design flexible, biocompatible composite implants by using a metal mesh as substrate and hydroxyapatite coating as bone regenerative stimulant derived from a simple sol–gel method. Experiments were carried out to understand the effect of the following parameters on implant’s performance (i.e., coating microstructure and adhesion, stiffness, hardness, biocorrosion, biocompatibility, and electrochemical behavior): (1) sol–gel method (dip-coating and drop casting), (2) substrate material (titanium and stainless steel), and (3) substrate mesh characteristics (mesh size and weave pattern). Although stainless steel implants are not commonly in use today, they have potential as coated biocompatible materials, as their regeneration time is higher than titanium or titanium alloys because they can possess a more stable passive layer.^13,23^ Furthermore, the mesh characteristics were expected to affect HA coating and ECM coverage based on open areas and wire waviness patterns. After mechanical testing through nanoindentation on the flexible HA/metal mesh samples, biocompatibility tests were performed to observe adipose-derived stem cells (ASCs) attachment, proliferation and osteogenic differentiation. Finally, electrochemical behavior and protective effect of HA coating were assessed through potentiodynamic polarization and impedance spectroscopy tests. According to our results, HA-coated titanium grade 1 showed the best overall performance as a potential cranioplasty implant, but HA-coated stainless steel with the finest mesh size could constitute an adequate alternative as human stem cells differentiated to bone cells and ECM through the mesh open areas after 21 days. Therefore, this flexible implant shows promise as a low-cost, customizable cranioplasty implant with potential for 3D ECM network and bone regeneration.

## 2 Materials and Methods

### 2.1 Materials

Potassium dihydrogen phosphate EMSURE ISO (KH_2_PO_4_) was supplied by Merck (Darmstadt, Germany); calcium nitrate tetrahydrate ≥99.0% (Ca(NO_3_)_2_.4H_2_O), ammonium hydroxide solution, and ASC reagent 28.0–30.0% NH_3_ basis (NH_4_OH) were purchased from Sigma-Aldrich (USA). Technical grade distilled water was purchased from ChemWorld (USA). Acetone AR ACS (C_3_H_6_O) was supplied by Macron Fine Chemicals, Avantor (USA). Ethyl alcohol, 95% denatured lab grade (C_2_H_5_OH), was bought from Aldon Company (USA).

Two mesh substrate materials (stainless steel and titanium), two wire diameters, and two mesh sizes were investigated. Stainless steel (ss) 304 and 316 mesh cloths, plain weave with 0.1 and 0.04 mm wire diameter, were purchased from McMaster-Carr (USA). The mesh sizes were 100 (i.e., 100 openings per 25.4 mm) and 200 (i.e., 200 openings per 25.4 mm) with 30% and 46% open area, respectively. Titanium mesh grade 1, twill weave with 0.1 mm wire diameter (mesh size 100), was supplied from Stanford Advanced Materials (USA). White titanium mesh (titanium + titanium oxide, brookite), twill weave with 0.1 mm wire diameter (mesh size 100), was acquired from Deze Wire (China). These mesh sizes and wire diameters were selected because of their flexibility and ease of cutting with scissors. All materials were used as received.

### 2.2 Synthesis of Hydroxyapatite Sol

Hydroxyapatite (HA) sols were prepared by mixing two precursor solutions to maintain Ca/P ratio as 1.67. First, 0.0167 mol of Ca(NO_3_)_2_·4H_2_O^15^ was dissolved in 50 mL of distilled water; 2.5 mL of NH_4_OH was added dropwise to the solution to adjust pH around 12, while stirring for 30 min. Second, a solution was made by adding 0.01 mol of KH_2_PO_4_ in 50 mL of distilled water and stirred for 30 min. Then, the second solution was added to the first one dropwise while stirring for another hour. The sol preparation was held at room temperature. Finally, 100 mL of HA white sol (pH ≈ 9) kept in glass container at room temperature for a week. This sol was used as general HA sol (GHA), referred to in the next paragraphs. A condensed HA sol (CHA) was derived from general HA sol. The general sol separated to transparent and white phases after aging for a week. The transparent solution was sucked by pipet to get a condensed HA white solution (pH ≈ 11).

### 2.3 Sol–Gel Coating Procedure and Samples Coding

Prior to coating, all samples were degreased by soaking in acetone, dried in air at room temperature for 5 min, and then, dried in an oven at 65 °C for 15 min. Each sol was sonicated for 15 min to get a homogeneous solution before the coating process. An area of 1.5 cm^2^ on each substrate was coated by two dissimilar separate sol–gel processes to assess the best coating procedure. Samples with one, two, and three layers of coating were made to determine the effect of multilayered films on implants characterization.

Two coating procedures were investigated in this study: dip-coating and drop casting. For dip-coating sol–gel (withdrawn rate = 20 000 μm/min, immersion time = 10 s), the sol was general HA (GHA) for one group and condensed HA (CHA) for another group. For multilayer coating, the substrate was kept in air for 30 s between two immersions until reaching the desired number of HA layers. For drop casting, a 1.5 cm^2^ area, on each side of the substrate, was covered with GHA by eye dropper. The adhesion of the liquid sol was sufficient so that the sample could be flipped and the other side could be covered by sol as well. For multilayer coating, the substrate was dried in an oven for 1 h at 150 °C and cooled in the oven to room temperature between each coating until reaching the desired number of HA layers. All samples were dried at room temperature, 50% humidity, immediately after immersion, then calcined in an oven at 150 °C for 1 h. Samples for all characterization and biocompatibility tests were prepared at the same time. The samples were kept at room temperature, in Petri dish in a dark place.

For future reference in the subsequent sections of this manuscript, sample coding was done in accordance with substrate material, mesh size, coating solution and procedure, and number of HA layers. Definition and examples are represented in Table 1.

**Table 1:** Samples Coding with Definition of Terms Used in This Manuscript

sample code example	substrate material	mesh size	coating solution and procedure	number of layers
ss304.100.DC3	stainless steel 304 (ss304)			1, 2, 3
ss304.200.GS1				
ss316.100.DC1	stainless steel 316 (ss316)		DC (drop cast from GHA)	
ss316.200.CS2		100	GS (sol–gel dip-coating from GHA)	
Tig1.100.GS2	titanium grade 1 (Tig1)	200	CS (sol–gel dip-coating from CHA)	
WTi.100.CS1	white titanium (WTi)		b (uncoated bare substrate)	
WTi.100.b				

### 2.4 Chemical Composition and Phase Analysis

X-ray diffraction (XRD) patterns were obtained for HA powders derived from three methods: (1) from HA coating powders, collected from both sides of HA-coated titanium samples by scratching the coating off with a razor blade (coatings from GHA and CHA sols, dried in an oven for 1 h at 150 °C); (2) from GHA sol dried at 700 °C in a glass beaker for 1 h to compare our data with literature and the coating powders from method 1; and (3) from GHA sol after aging for 1.5 year to analyze the crystal structure and stability of the solution after a long storage time. An X-ray diffractometer (PANalytical Empyrean) over 2θ range of 5–90° was used to determine crystal structure for all powders. The process operated at continuous CuKα radiation (λ = 0.1540598 nm) with a step size of 0.02°, generator voltage of 45 kV, and tube current of 40 mA. Energy-dispersive X-ray spectroscopy (EDX) with field emission electron source (FEI QUANTA 3D FEG FIB/SEM) using an accelerating voltage of 20 kV in statistical imaging mode was used to determine Ca/P ratio in HA coating on substrate. HA crystallographic structure and the most brilliant plane spaces and indices were studied by high-resolution transmission electron microscopy (HR-TEM, JEOL JEM-2011) equipped with a bottom-mounted Gatan SC1000 CCD camera with an accelerating voltage of 200 kV.

### 2.5 Microstructure and Morphology

The morphology of HA coating on the mesh substrates was analyzed by focused ion beam (FIB) with a high-resolution field emission gun scanning electron microscope (FE-SEM, FEI QUANTA 3D FEG FIB/SEM) using an accelerating voltage of 5 and 20 kV. Images were also captured before and after polarization tests to determine the corrosive effects of simulated body fluid (SBF).^24^ Thickness of HA layers was measured from tilted images or cut sections made by FIB. Effect of coating method and number of HA layers on mesh coverage was further analyzed by quantifying the following: coverage of mesh wires (in % value with respect to total mesh area) and open mesh area (in % value with respect to total mesh area). An image analysis software (Image J, National Institutes of Health) was used to calculate mesh coverage for three to five samples (*n* = 3–5) for each coating method/HA layer combination. An example of image analysis with uncoated wire area and open mesh areas is shown in Figure S1.

### 2.6 Hardness and Modulus of Elasticity

To determine resistance to surface deformation and stiffness of HA coating on wires of the mesh substrates, nanoindentation hardness measurements were conducted on the top surface of the wires. After several preliminary trials, it was determined that samples with the thickest HA coating (DC3) were the most suitable to carry out nanoindentation tests, as it prevented the nanoindenter tip from slipping and drafting on the wires’ curved surface. All samples were glued to a flat, hard surface before nanoindentation. The tests were also carried out on bare substrates as a reference. All hardness measurements were performed with a Nanoindenter XP system (MTS Systems Corp., Knoxville, TN), in a force-controlled mode with a maximum force of 10 mN and a force rate of 0.7 mN/s. For each sample, six testing points were collected, and the distance between each two adjacent points was set to 15 μm.

### 2.7 Biocorrosion and Biocompatibility

#### 2.7.1 Hank’s Salt Solution Immersion Test

Biocorrosion test was performed by immersion in Hank’s salt solution^14,25^ for all HA-coated mesh samples (all conditions defined in Table 1) for 48 h at 37 °C. The sustainability and morphology of samples in marked areas were examined by optical microscopy (Meiji Techno MT8100F) before and after immersion in Hank’s salt solution.

#### 2.7.2 Cell Culture

Biocompatibility for all HA-coated mesh samples (all conditions defined in Table 1, for a total of 54 samples) was studied by cell culture in well plates. A detailed timeline of all experiments and samples selected for each step related to cell culture (section 2.7.2), cytotoxicity determination (section 2.7.3), osteogenic differentiation (section 2.7.4), and immunocytochemistry (section 2.7.5) is shown in Figure S2. Figure S3 shows a summary of all initial sample conditions based on mesh substrate, mesh size and coating procedure. Human adipose-derived stem cells (ASCs) frozen at passage 0 (P0) were supplied by LaCell LLC (New Orleans, LA, USA). The vials were thawed in a water bath at 37 °C for 2 min and diluted with stromal medium (89% dulbecco’s modified eagle medium (DMEM), 10% fetal bovine serum (FBS) and 1% anti–anti 100× antibiotic–antimycotic, all from Gibco) to remove cryoprotectant agent. Then, the samples were centrifuged at 1500 rpm for 5 min to obtain cell pellets and the supernatant was removed. The cell pellets were resuspended in stromal media and cultured until passage 3 (P3) in T flasks in incubator at 37 °C and 5% CO_2_ and 95% humidity as described in literature.^26−28^

#### 2.7.3 Cytotoxicity Determination

Bioactivity for all bare substrates and HA-coated samples was measured by means of viability after 72 h of direct contact between ASCs and samples in 12-well plates. The test conditions were modified based on ISO 10993-5 (Biological evaluation of medical devices, Part 5: Tests for invitro cytotoxicity). Viability test was also carried out for control and test groups after 35 days (as described in section 2.7.4 below and Figure S2) to show the effect of long-time exposure of ASCs and osteogenic differentiated ASCs to the metallic materials. Cell viability was measured by trypan blue exclusion assay,^29^ as other assays based on absorbance measurements (optical density, O.D.) were not accurate for metal samples. All experiments were performed for three samples (*n* = 3), and the results are reported for bare substrates (six samples) and the test group (six samples).

#### 2.7.4 Osteogenic Differentiation

Prior to placing uncoated and coated mesh samples in well plates, they were immersed in ethanol (95%) for 72 h, then dried, and placed under UV light for 3 h in a biological safety cabinet. ASCs were transferred from T flask to 12-well plates. Each well contained one sample and 1.5 × 10^4^ cells were placed on the sample’s top surface in the well. Two control groups and one test group were defined for osteogenic differentiation tests. The first and second control groups were uncoated substrates (one for each mesh substrate material type) covered by ASCs in stromal medium. The initial study group was comprised of all samples covered by ASCs in 12-well plates in stromal media. Well plates were kept in an incubator at 37 °C and 5% CO_2_ and 95% humidity during cell culture. The medium was refreshed every 3 days for all samples. All samples were moved to a new well plate after 7 and 14 days to keep cell proliferation only on the samples’ surface. After 14 days, the samples with the most cells on both sides were chosen for each substrate type as the actual test group (i.e., six samples, one for each mesh substrate material type). Then, this new test group and the second control group were placed in a new 12-well plate and an osteogenic differentiation medium (Obatala Sciences, New Orleans, LA, USA) was added to it for 21 days. The first control group was continued for 21 days in stromal media in this stage.

After day 21, a part of each sample was cut and fixed for FE-SEM imaging to study the morphology and microstructure of the formed tissue layer on the sample, while the other cells were trypsinized for TEM (JEOL JEM 1400 TEM, 120 kV) and viability tests. Cell viability was measured by trypan blue exclusion assay.^29^ ECM coverage on the samples was quantified in % value with respect to total area through image analysis (Image J, National Institutes of Health). All measurements were acquired for three samples (*n* = 3).

#### 2.7.5 Immunocytochemistry (ICC)

As the ASCs underwent osteoblast differentiation while feeding with osteogenic differentiation medium, the bone differentiation was validated with RUNX2 and Osteopontin (OPN) genes. RUNX2 is a transcription factor induced with bone differentiation to an osteogenic lineage. It is used to direct the osteoblast and is expected to be high in the beginning of the osteogenic lineage (<14 days). Osteopontin aids in attachment of osteoclast but is not expressed in osteoclast and is used to verify mineralized bone. This gene (OPN) is mostly detectable at the end of an osteo lineage (≤21 days). It is to be noted that when a gene is detected, it can exhibit variability as different stem cells from various donors will not be induced at the same rate.^30^

Immunocytochemistry was done on the surface of two samples among 12 HA-coated samples (those from the test group and second control group that had the lowest and highest ECM coverage after 21 days, identified in Figure S3) and one blank well plate (ASCs only) in osteogenic differentiation medium. Using primary and secondary antibody for RUNX2 and OPN, the expression of antigen on the scaffold (mesh samples) and blank well plate was visualized for 7, 14, and 21 days of cell culture based on manufacturer protocol. RUNX2 Polyclonal Antibody with Alexa Fluor 647 goat antirabbit IgG (H+L) and Osteopontin Monoclonal Antibody with Alexa Fluor 594 goat antimouse IgG (H+L) were used to detect osteoblast markers on the mesh samples. The nucleus and cytoskeleton of cells were dyed by Hoechst 33342 Solution (20 mM) and Phalloidin, DyLight 488, respectively. Thirty minutes after adding the last dye (Hoechst), samples were removed from the solution, washed with warm phosphate buffer saline (PBS) three times, and then, imaged using an inverted fluorescence microscope (Nikon Eclipse Ti2) and NIS Elements Advanced Research Microscope Imaging Software (NIS Elements AR, Nikon). All materials were supplied by Invitrogen, Life Technologies Corporation, USA.

### 2.8 Electrochemical Behavior Analysis

To examine the corrosion behavior and the protective effect of HA coating of the chosen samples after the biocompatibility tests and to evaluate the differences between coated and uncoated samples in human body medium, potentiodynamic polarization measurements were performed. A CHI 604C electrochemical workstation in simulated body fluid (SBF)^24^ with a standard tree-electrode corrosion cell set up was used. HA-coated or uncoated sample with a surface area of 1 cm^2^ was used as the working electrode, a platinum wire as the counter electrode and a saturated calomel electrode (SCE) as the reference electrode. Polarization curves were assessed by sweeping the potential from −0.6 V_SCE_ to +0.6 V_SCE_ at a scanning rate of 1.67 mV/s at room temperature.^31^ All samples were soaked in SBF solution around 1 h before potentiodynamic polarization tests to stabilize the open circuit potential (OCP). Electrochemical impedance spectroscopy (EIS) was performed to analyze the electrochemical behavior with low frequency of 0.01 Hz, high frequency of 10000 Hz, and an amplitude of 0.05 V. Nyquist and bode plots were sketched according to EIS results and an equivalent circuit was selected based on literature review of the same substrate material type (Ti or SS).^32^ All experiments were reported for three samples (*n* = 3) to minimize the error.

### 2.9 Statistical Analysis

In the Results and Discussion section, data are represented as the mean ± standard deviation (SD). Two-way analysis of variance (ANOVA), followed by Tukey’s multiple comparison test, was used to compare HA and ECM coverage data at a significant level of *p* < 0.05. For biocompatibility test results, the difference between control groups and the samples (test group) was determined through two-tailed Student’s *t* test at the significant level of *p* < 0.05. On the bar graphs, because of the large number of different samples, the statistical significance of a limited number of pairs is indicated for clarity. Details regarding statistical analysis are provided in the figures’ captions.

## 3 Results and Discussion

### 3.1 Chemical Composition and Phase Analysis

As explained in section 2.4, HA powders were collected from both sides of HA-coated titanium samples by scratching the coating off with a razor blade. Different samples were scratched off for drop casting and dip-coating methods with GHA- and CHA-based coatings, dried in an oven for 1 h at 150 °C. HA powder samples were also made from GHA sol at 700 °C for 1 h in a glass beaker to compare our data with references for higher temperature calcination, and to confirm the lower temperature-made coating powders had the same crystal structure as higher temperature-made powders. A fourth powder type was made from GHA after aging for 1.5 year to analyze the crystal structure and stability of the solution after a long storage time.

**Figure 1:**
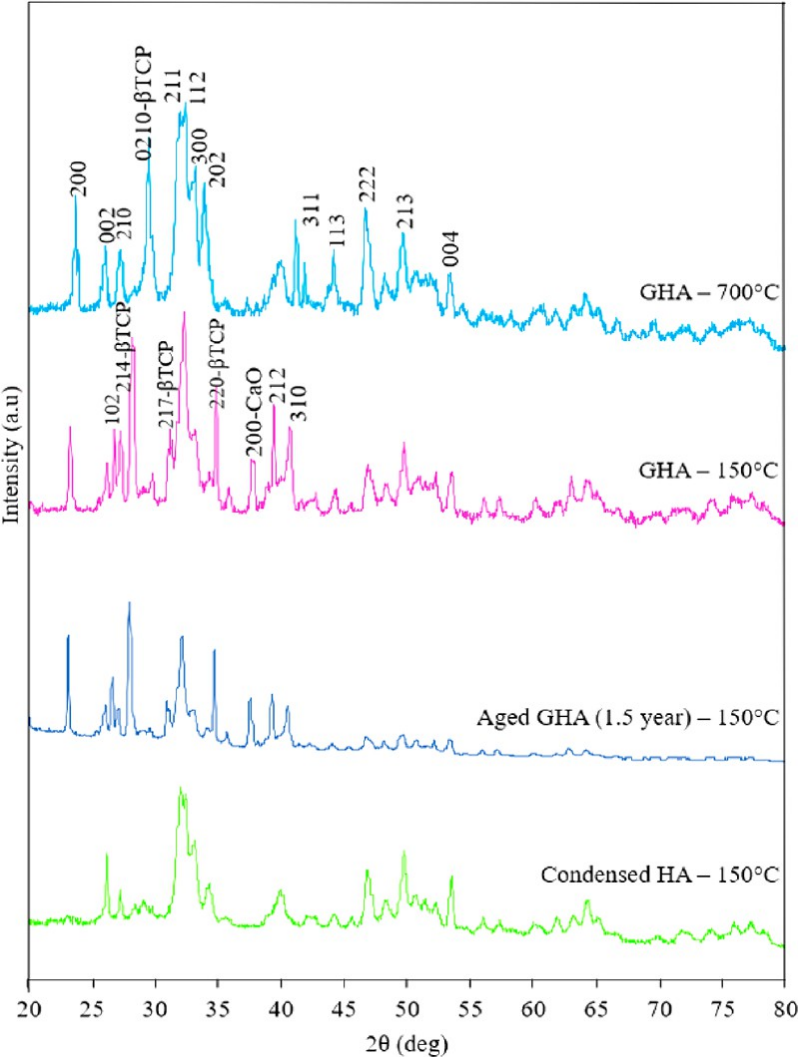
XRD patterns for the following powders (from top to bottom): general hydroxyapatite sol (GHA) dried at 700 °C, scratched off coating powder made from GHA sol dried at 150 °C, aged GHA sol (1.5 year old) dried at 150 °C, and scratched off coating powder made from condensed hydroxyapatite sol (CHA) dried at 150 °C.

X-ray diffraction patterns for all HA powders are shown in Figure 1. A pure crystalline hydroxyapatite phase is observed from the characteristic peaks for CHA powder, consistent with standard database (JCPDS 09-9432). For HA powders from both coated and aged GHA samples, diffraction patterns mostly show the peaks for crystalline pure HA, but also for decomposed compounds of HA, β-tricalcium phosphate (214, 217, 220) (β-TCP) and CaO (200). Characterization peaks for both powders are the same, which indicates stability of the general HA solution during long storage time (1.5 year). The diffraction pattern for GHA samples dried at 700 °C shows both pure hydroxyapatite and β-tricalcium phosphate (0210). It is observed that the structure for GHA powders made at 150 and 700 °C is the same, but differences about the direction of some β-TCP and CaO planes make them undetectable in XRD patterns. As the GHA solution is calcium-deficient hydroxyapatite, which is unstable against thermal treatment with a pH ≈ 9, calcination induced decomposition of the structure into three different calcium phosphate phases: pure HA, β-TCP, and CaO.^33^ CHA solution was a precipitated solution and had a pH around 11, which is above 10 and preferable to produce pure HA, as observed in Figure 1.^34^ Since all samples were dried at temperatures lower than 1000 °C, β-tricalcium phosphate is most likely to be formed for powders from GHA sol.^35^ Overall, crystal structure and characteristic peaks for powders collected from drop casting and dip-coating methods are the same. For our research, no issue is expected with mixed structures of hydroxyapatite and β-tricalcium phosphate (biphasic hydroxyapatite) for bone regeneration purposes as tricalcium phosphate is a better material to stimulate bone growth.^36^

**Figure 2:**
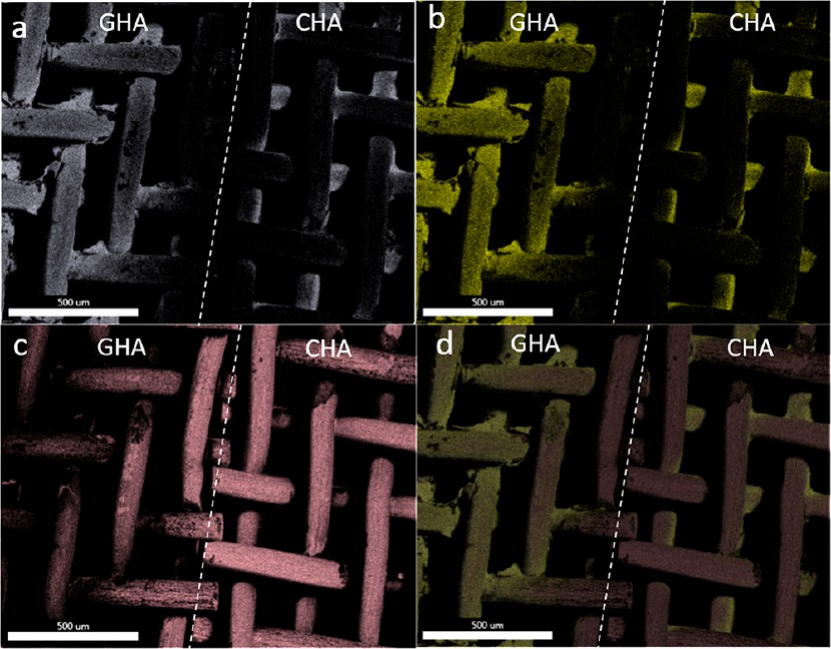
Representative FE-SEM mapping images (EDX) of elemental distribution for Ca and P in one layer HA coatings derived from GHA and CHA solutions by dip coating method, on titanium grade 1 (Tig1) mesh substrates: (a) Ca, (b) P, (c) Ti, and (d) merge mapping for GHA (left) and CHA (right). Scale bar is 500 μm.

EDX results for elemental distribution of Ca and P are presented in Figure 2a and b, respectively. The Ca/P ratio was 1.67 for powders from CHA solution and 1.86 for powders from GHA. However, in GHA powders, there are three different compounds: HA, β-TCP, and CaO. As those all contain calcium, it is difficult to find the accurate Ca/P ratio for HA in this powder. EDS mapping images for one layer HA coatings derived from GHA and CHA solutions by dip coating method on titanium grade 1 (Tig1) showed a homogeneous dispersion of calcium (Figure 2a). Figure 2c and d displayed the visible substrate (Tig1, pink colored areas) in mapping pictures, indicating uncoated areas.

**Figure 3:**
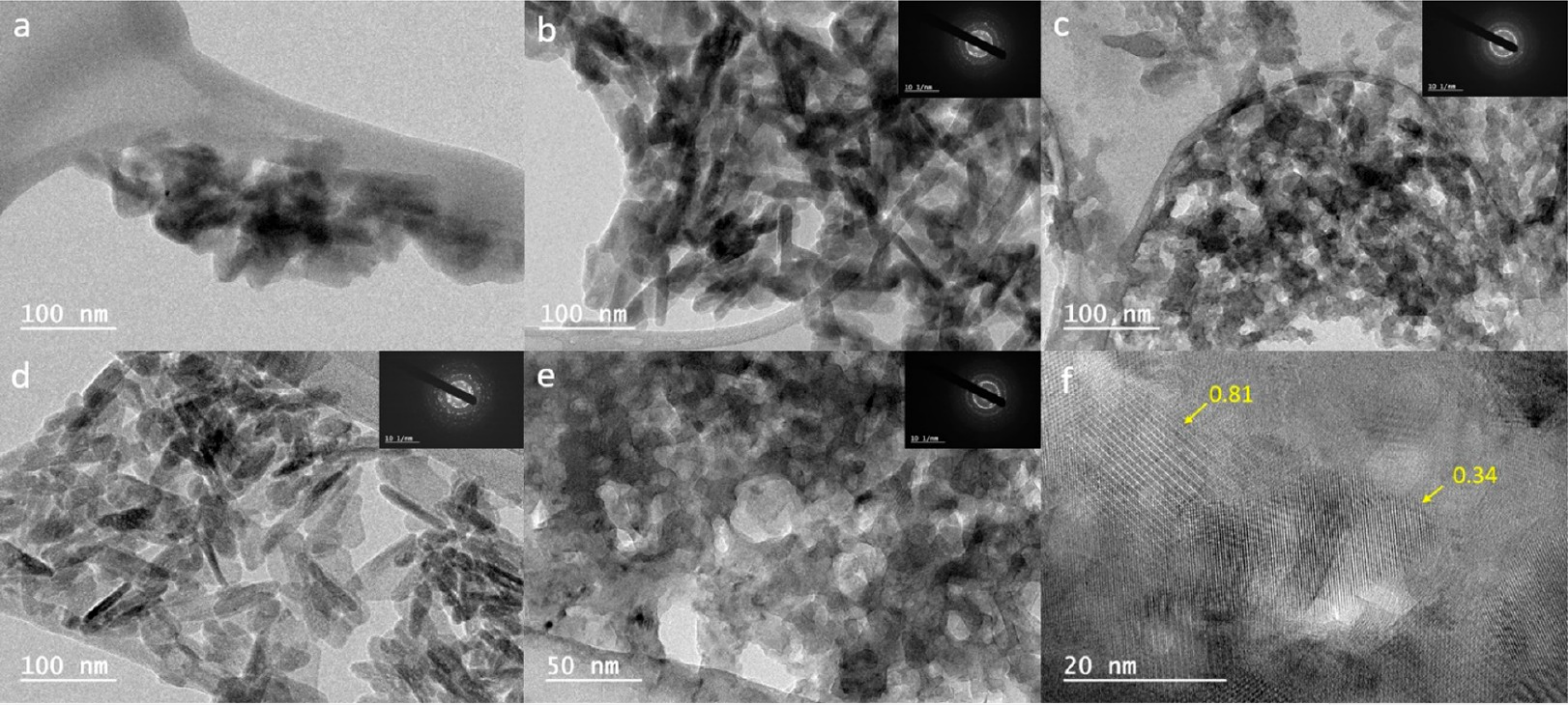
Representative HR-TEM images for coating powders (scratched off from substrate surface) derived from different sols, dried at various temperatures: (a, b) GHA, (c) aged (1.5 year) GHA, (d) CHA dried at 150 °C, and (e, f) GHA dried at 700 °C. Pure HA plane spaces of *d* = 0.81 nm (001) and *d* = 0.34 nm (002) are marked in panel f. Scale bar is 100 nm for (a, b, c, d) and 50 nm for (e) and 20 nm for (f). Images in inset are related to diffraction patterns with a scale bar of 10 nm^–1^.

HR-TEM pictures were captured to characterize the nano crystal shape of HA powders and interplanar spacing of the most intense reflections (Figure 3). Figure 3a and b shows HA crystals in two different directions for GHA powder, with aged GHA in Figure 3c. HA nanorods are visible in Figure 3b and d with lengths from 30 to 50 nm and diameters from 10 to 15 nm. Figure 3e and f reveals the structure of GHA powder dried at 700 °C at two different magnitudes, which presents pure HA plane spaces equal to *d* = 0.81 nm (001) and *d* = 0.34 nm (002).^37^ Those planes were observed for all powder samples. Different coating procedures and numbers of layers did not affect crystal structure, shape or most intense plane reflections.

### 3.2 Microstructure and Surface Morphology

**Figure 4:**
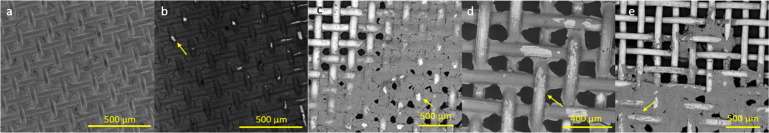
Representative FE-SEM images in secondary electron (SE) and backscatter electron (BSE) modes for examples of 3 layered HA coatings derived from drop casting method (DC3) on (a) ss304.200 (SE), (b) ss304.200 (BSE), (c) ss304.100 (BSE), (d) Tig1.100 (BSE), and (e) WTi.100 (BSE). Scale bar is 400 μm in panel d and 500 μm in the rest. Yellow arrows indicate uncoated wire peaks.

**Figure 5:**
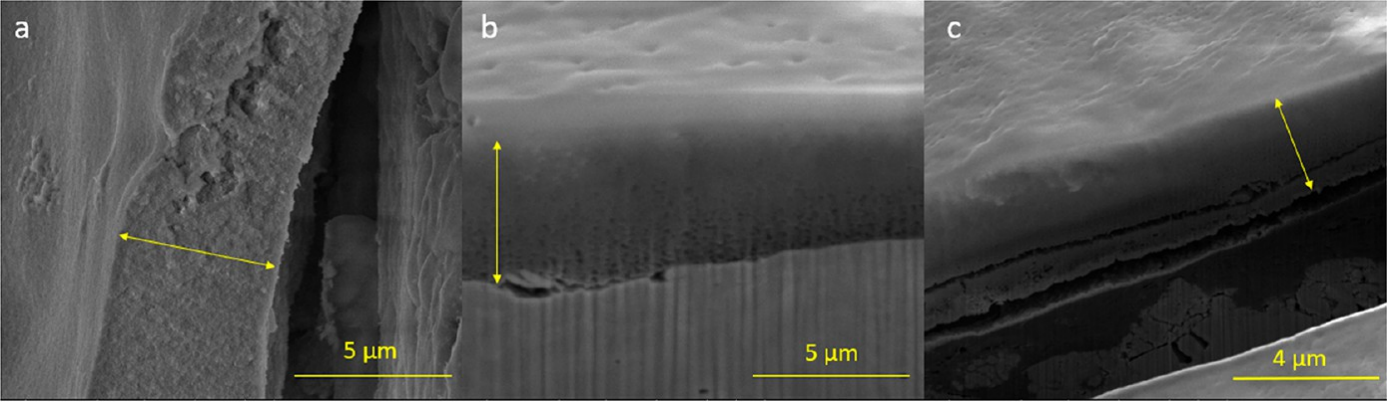
Representative FE-SEM (SE) images of delaminated HA coating and FIB cross sections to measure thickness of HA coating with three layers derived from drop casting method on (a) ss316.200, (b) ss316.100 (tilted at 52°), and (c) Tig1.100 (tilted at 52°). Yellow arrows indicate the HA coating. Scale bar is 5 μm in panels a and b and 4 μm in panel c.

**Figure 6:**
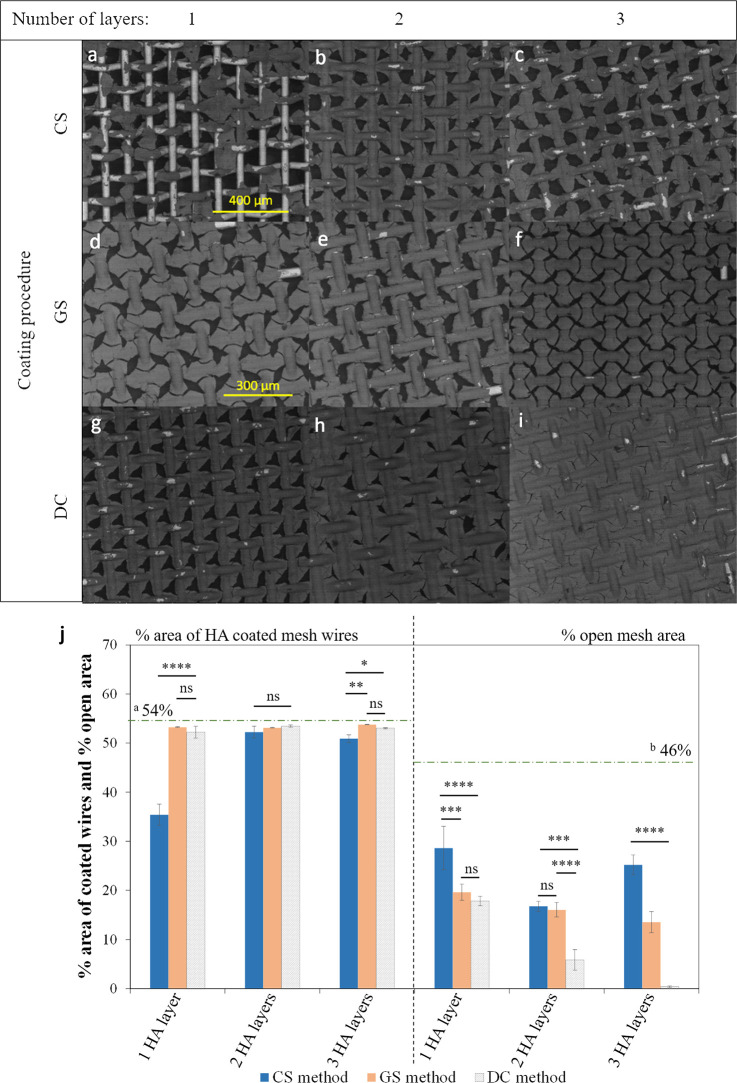
Representative FE-SEM (BSE) images for ss304.200 substrates with different numbers of HA layers, derived from different solutions (GHA, CHA) and procedures (CS, GS, DC). (a, b, c) CS, substrates coated by sol–gel dip-coating with CHA sol with 1, 2, and 3 HA layers; (d, e, f) GS, substrates coated by sol–gel dip-coating with GHA sol with 1, 2, and 3 HA layers; and (g, h, i) DC, substrates coated by drop casting with GHA sol with 1, 2, and 3 HA layers. Scale bar is 400 μm in all except panels d and e, which is 300 μm. (j) Corresponding average % area of HA-coated mesh wires and open mesh areas for all coating procedures and number of HA layers (compared to manufacturer’s values, ^a^54% and ^b^46%). (ns = not significant, *p < 0.05, ***p < 0.001, ****p < 0.0001, *n* = 3.)

FE-SEM images were used to analyze the effect of substrate material, mesh size, number of HA layers, and coating solution and procedure on microstructure, uniformity and coverage of HA coating on the substrates (Figure 4 to Figure 6). Before investigating the effect of the coating procedure and number of HA layers in detail, coating quality for different substrate materials and mesh sizes was first qualitatively assessed for cracks and adhesion (Figure 4 and Figure 5). Representative FE-SEM images in back scatter and secondary electron modes (BSE and SE, respectively) were captured. However, as images in BSE mode helped differentiate between coating and substrate, as they both displayed similar gray scale in SE mode, they were used for most observations and image analyses for all samples. A comparison between those two modes is shown in Figure 4a,b.

#### 3.2.1 Effect of Substrate Material and Weave Pattern

The substrate material is a factor affecting adhesion and coverage of HA coating. Weaving pattern for mesh samples is another important factor impressing uniformity of coating on the surface and interface cohesion between coating and substrate. Examples of HA coating with three layers derived from drop casting method (DC3) are represented in Figure 4 as they are most representative for this discussion. The discussed pattern was observed for other coating solutions, procedures and number of layers on all materials. Figures 4b and c and 5b show that stainless steel 304 and 316 with plain weave led to an overall more uniform coverage and adhesion of HA coating on different areas of the wire when compared to titanium samples with twill weave (Figures 4d, e and 5c). It is expected that the different bending ratios of the wires in the two patterns (plain and twill weave for the same mesh size) impacted distortion and internal stresses in HA coating, leading to uncovered areas or interfacial gaps located, in particular, at the wire peaks on titanium mesh samples (yellow arrows in Figure 4d, e and gap in Figure 5c). Moreover, based on the TiO_2_ chemical structure and its tendency for physical or chemical bonding with calcium or phosphate ions in HA,^38^ adhesion of HA coating on pure titanium (Tig1) substrates is likely lower than titanium + titanium dioxide (WTi), as the HA coating completely covered some open areas in WTi samples, but not in Tig1 samples (Figure 4d, e). The microstructure of HA coating was similar in all samples, featuring a smooth appearance on the wires’ surface, but with microcracks between wires or delamination seen on cross-sectional images (Figure 5). Overall, the microstructure of HA coating was not affected by weave pattern or substrate material.

#### 3.2.2 Effect of Mesh Size

Regarding the effect of mesh size (100 and 200), higher mesh size (200 with wire diameter *d* = 0.04 mm for ss304 and ss316 substrates) led to more uniform coverage compared to substrates with lower mesh size (100) (Figure 4b, c). Higher mesh size substrates have thinner wires and smaller holes, but overall more open area (46% vs 30%). It is expected the latter can promote solution’s movement from one side to the other side of the substrate during coating, which leads to more contact between solution and wire’s surface, resulting in more uniform coverage. The topography of the coating surface for mesh size 200 (smaller wire diameter) is smoother than mesh size 100 due to the overall flatter pattern: reduced wire bending at the junctions and smaller distance between wire peaks and valleys.^39,40^

#### 3.2.3 Coating Thickness

Increasing the number of HA layers on the substrates increased coating thickness and improved coverage. However, it also multiplied cracking and delamination of HA coating, as thicker coating may crack and detach easier than thinner one. Table 2 summarizes the measured maximum thickness from the thickest coating (DC3 samples) on all substrate materials by FIB cross-section or coating delaminated edge in FE-SEM pictures (Figure 5). As the coating coverage was not uniform, the HA coating thickness on each substrate varied from none up to the reported maximum thickness. For stainless steel samples, the maximum thickness was 5.4 ± 0.9 μm, while it was 4.0 ± 0.6 μm for titanium grade 1 and 7.9 ± 0.9 μm for white titanium. The highest standard deviation value (0.9 μm) is related to the variations induced by the cylindrical shape of the wires, wire junctions, and pattern waviness. All thickness values were in the same range as reported in the literature for sol–gel-derived HA coating on different substrates (0.07–9 μm).^41^ The highest coating thickness value was obtained for white titanium, which contains titanium dioxide (TiO_2_). As stated in section 3.2.1, adhesion of HA on TiO_2_ is higher than Ti,^42^ thus the higher thickness of HA layers on WTi samples.

**Table 2:** Thickness Measurements (Maximum Values) Based on FE-SEM Pictures for All Substrates with Three Layers of HA Coating Applied by Drop Casting Method from GHA Solution (Average ± Standard Deviation, *n* = 9)

sample code	ss304.100.DC3	ss304.200.DC3	ss316.100.DC3	ss316.200.DC3	Tig1.100.DC3	WTi.100.DC3
thickness (μm)	5.4 ± 0.9	5.4 ± 0.9	5.4 ± 0.9	5.3 ± 0.6	4.0 ± 0.6	7.9 ± 0.9

#### 3.2.4 Effect of Coating Solution, Procedure, and Number of HA Layers

Figure 6 illustrates a representative summary of the effect of coating procedure, solution type, and number of HA layers on mesh coverage for ss304 mesh size 200 substrates (ss304.200). For any constant mesh size, similar coverage behavior was observed for all other substrate materials (ss316, Tig1, and WTi). Figure 6j shows the average % area of coated mesh wires and % open mesh area for all conditions shown in Figure 6a–i.

GHA and CHA solutions exhibited different viscosities, which affected HA film coverage (Figure 6j) and uniformity on the substrates. CHA solution (CS row, Figure 6a–c) exhibited higher viscosity and consequently, the solution dragged the liquid on the substrate while the sample was moved out during the dip-coating process. Thus, overall, the coverage of the GHA solution (GS row, Figure 6d–f), which is above 50% area coverage on wires and less than 20% open areas (Figure 6j), was better than CHA (CS row) on all substrates with different materials and mesh sizes. The microstructure of the HA coating derived from various HA solutions was not significantly affected as their chemical composition and crystal structure are similar (see section 3.1). The dip-coating process (CS and GS rows), compared to drop casting (DC row, Figure 6g–i), led to more uniform films and coverage on the wires, as this process is homogeneous and controlled during the coating procedure. There was no excess solution on the substrates after the coating process or before drying. The drop casting process provided coatings with higher thickness values with microcracks, as the excess solution remained on the substrates and was dried in the oven. This led to high wire coverage with sealed holes in some areas (less than 1% open area with more than 52% wire coverage), while wires remained uncovered in other areas.

By analyzing images of HA coating with various number of layers (1–3 in Figure 6), for the dip-coating process (CS and GS rows), it can be assumed that the first layer coverage depends on solution viscosity. The lower viscosity of the GHA solution led to higher coverage and drying in air for 30 s was long enough to set the layers (GS method in Figure 6j). For the CS coating procedure, coverage of wires and open areas after the second layer was improved. The second layer smoothed out any sections of the first layer that had not been set or bonded to the substrate. Wire coverage after the third layer remained similar for both methods, but open areas exhibited a significant difference (Figure 6j). GS3 samples in particular displayed a uniform HA coating pattern (Figure 6f). For the drop casting method (DC row), the samples were dried in oven before adding the next layer. Adding second and third layers increased the thickness and coverage, until the layer was too thick and delaminated from the substrate. Microcracks mostly appeared at wire junctions due to thermal mismatch between substrate/coating and internal stress in the coating. Delamination occurred at the wire peaks, resulting from the coating internal tension related to the bent shape of the wires. In general, for all substrate materials, the highest coverage and thickness were obtained for DC3 samples, the most uniform coverage was observed for GS samples, and the lowest wire coverage was noted for samples CS1.

### 3.3 Hardness and Modulus of Elasticity

Hardness and modulus of elasticity are two important properties to determine mechanical performance of metal/ceramic implants. Modulus of elasticity of HA coating should be close to the modulus of natural skull bone (3.3–6.0 GPa) for cranioplasty applications. In addition, hardness should be high enough to bear induced mechanical load after implantation.^32,43^

**Figure 7:**
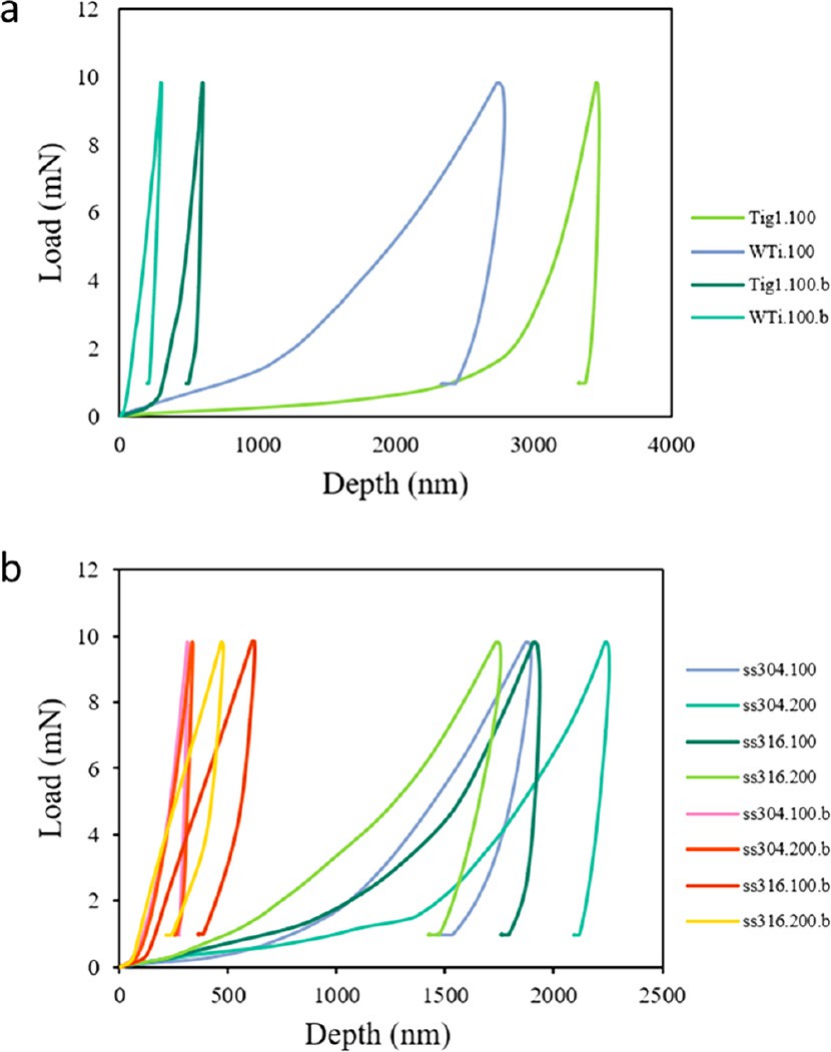
Load-indentation curves and release patterns for HA-coated samples (DC3) and bare substrates (.b) for (a) titanium and (b) stainless steel substrates.

Figure 7 and Table 3 show load-indentation curves for nanoindentation tests, as well as corresponding hardness and modulus of elasticity values. The inserting depth for bare substrates was around 500 nm, while it was more than 2500 nm for HA-coated titanium substrates and more than 1000 nm for HA-coated stainless steel substrates. Hardness values for HA-coated stainless steel substrates are higher than titanium ones as HA coating is more uniform (as seen in Figure 5b, c). The lowest hardness value (39.0 MPa) is more than double the highest value reported in the literature for HA coating on titanium substrates (17.5 MPa).^20^ Moduli of elasticity for all coated substrates were in the range of 2.2–18.3 GPa, close to reported values for natural human skull bone (3.3–6.0 GPa). On the other hand, the moduli of elasticity values for bare substrates were higher than natural bone. Thus, mesh substrates with HA coating are more similar to natural bone than bare metal ones. This is preferable to keep consistency between the implant and the surrounding bone. High standard deviation values in Table 3 are related to the topography of the surface (cylindrical shape of wire) and the drift of the nanoindenter tip. Flat surfaces generally present fewer variations in measurements.

**Table 3:** Hardness and Modulus of Elasticity Calculated from Figure 7 for HA-Coated (DC3) and Bare Substrates (Average ± Standard Deviation)

sample code (HA-coated, DC3)	hardness (MPa)	modulus of elasticity (GPa)	sample code (bare substrates)	hardness (GPa)	modulus of elasticity (GPa)
ss304.100	77.8 ± 35.5	3.7 ± 1.2	ss304.100.b	4.8 ± 0.6	137 ± 16.8
ss304.200	64.4 ± 31.4	5.9 ± 2.1	ss304.200.b	4.2 ± 0.1	191 ± 14
ss316.100	111.2 ± 32.4	18.3 ± 9.0	ss316.100.b	1.5 ± 0.2	114 ± 5
ss316.200	84.2 ± 41.8	3.2 ± 1.2	ss316.200.b	1.7 ± 0.4	36 ± 6
Tig1.100	54.0 ± 17.3	11.3 ± 2.1	Tig1.100.b	0.7 ± 0.2	64 ± 9
WTi.100	39.0 ± 18.1	2.2 ± 0.6	WTi.100.b	2.6 ± 2.5	71 ± 30

### 3.4 Biocorrosion and Biocompatibility

Before using ASCs for cell culturing, stability and biocorrosion behavior for all samples were determined by immersion in Hank’s salt solution at human body temperature (37 °C). Optical microscopy images for all samples before and after immersion for 48 h in Hank’s salt solution displayed the same morphology without any signs of coating delamination or dissolution in Hank’s solution. Coating with pure or biphasic hydroxyapatite composition was stable and displayed good adhesion after contact with body fluid solution at 37 °C.

ASCs were used based on their ease of application. They have also been used for craniofacial repair and regeneration based on a review of preclinical and clinical studies.^44^ Using optical microscopy with magnification up to 10×, one HA-coated sample (for each mesh substrate) with the most stem cells attached to the substrate was chosen and placed in new 12-well plates with osteogenic media for 21 days. For the test group, this resulted into the following sample selection: samples with one HA layer for stainless steel substrates and two HA layers for titanium substrates. All chosen samples were coated through the same method (dip-coating process, CS or GS). Thickness for these samples was approximately 3 μm. In the first and second control groups, the uncoated mesh for each substrate was transferred to a 12-well plate in stromal and osteogenic medium for 21 days, respectively.

#### 3.4.1 Cell Viability and Cell Proliferation

**Figure 8:**
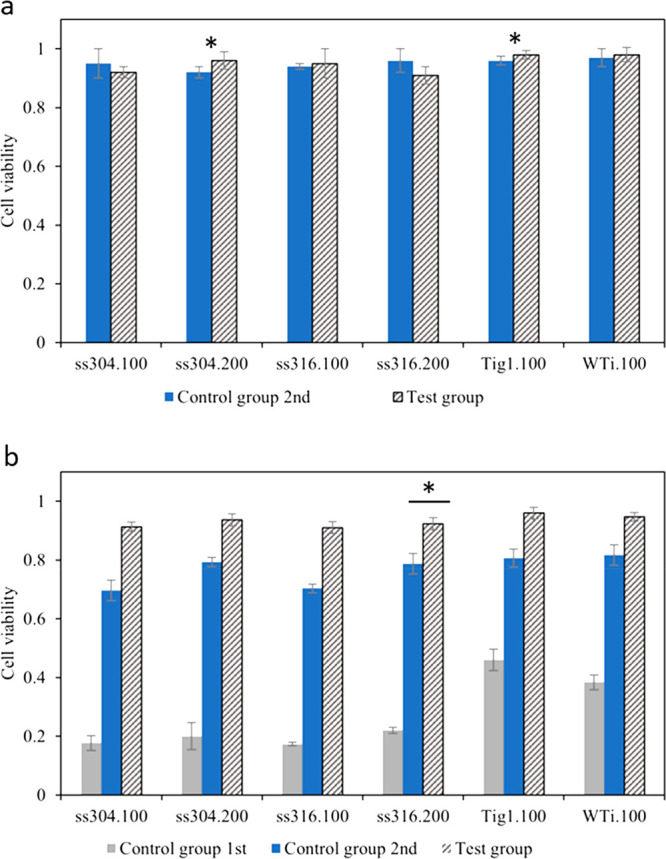
Cell viability after (a) 3 days and (b) 35 days calculated for two control groups and one test group. First control group: Uncoated substrates in stromal medium for 35 days. Second control group: Uncoated substrates in stromal medium for 14 days, then placed in new well plate with osteogenic differentiation medium for 21 days. Test group: HA-coated samples. Cell viability was measured using trypan blue assay. In panel a, *p < 0.05 for two pairs, not significant for all others (*n* = 3). In panel b, *p < 0.05 for one pair and **p < 0.01 for all others (*n* = 3).

For cytotoxicity analysis, cell viability was measured for uncoated substrates and selected HA-coated substrates (test group) after 72 h (3 days). The timeline was chosen based on the performance of the materials, during the first days, as there were no significant changes in well plates until 48 h. Figure 8a shows cytotoxic potential for uncoated/coated metal substrates is low as viability is more than 90% for the second control group (uncoated substrates) and test group (HA-coated substrates) in stromal media after 3 days. Cell viability was also calculated after 35 days for the first control group (uncoated samples in stromal media), and after 21 days for the second control group (uncoated samples in osteogenic media) and the test group (HA-coated samples in osteogenic media) at the end of the cell culture procedure. Figure 8b shows approximately up to 50% increase (0.5) in cell viability when using osteogenic media for control groups. Cell viability was more than 90% for all coated samples, which confirms HA coating can improve cell viability by up to 20% (0.2) in the same medium (difference between second control group and test group). The best average result (98%) was obtained for HA-coated Tig1, but all values remained within standard deviation, which suggests the material of the substrate marginally affects cell viability. There is a significant difference between uncoated and HA-coated substrates in stromal medium for ASCs and osteogenic medium for differentiated ASCs (bone cells) as *p* < 0.05 for all pairs in Figure 8b.

**Figure 9:**
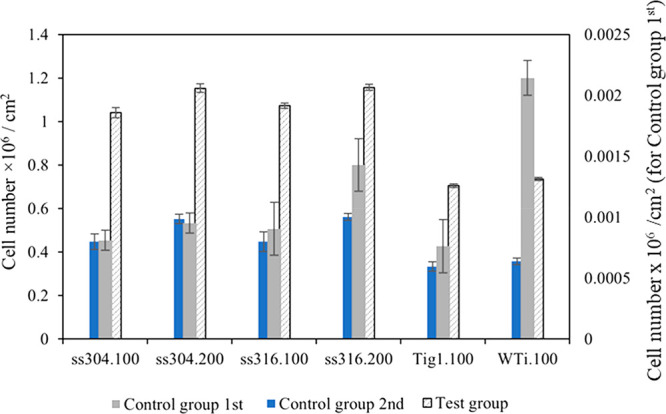
Cell numbers per substrate cm^2^ after 35 days, counted for cells collected from both sides of mesh samples in two control groups and one test group. First control group: Uncoated substrates in stromal medium for 35 days. Second control group: Uncoated substrates in stromal medium for 14 days, then placed in new well plate with osteogenic differentiation medium for 21 days. Test group: HA-coated samples. All test group results are significant compared to both control groups (***p < 0.001, *n* = 3). Cell numbers were measured using hemocytometer (mean ± SD, *n* = 3).

**Figure 10:**
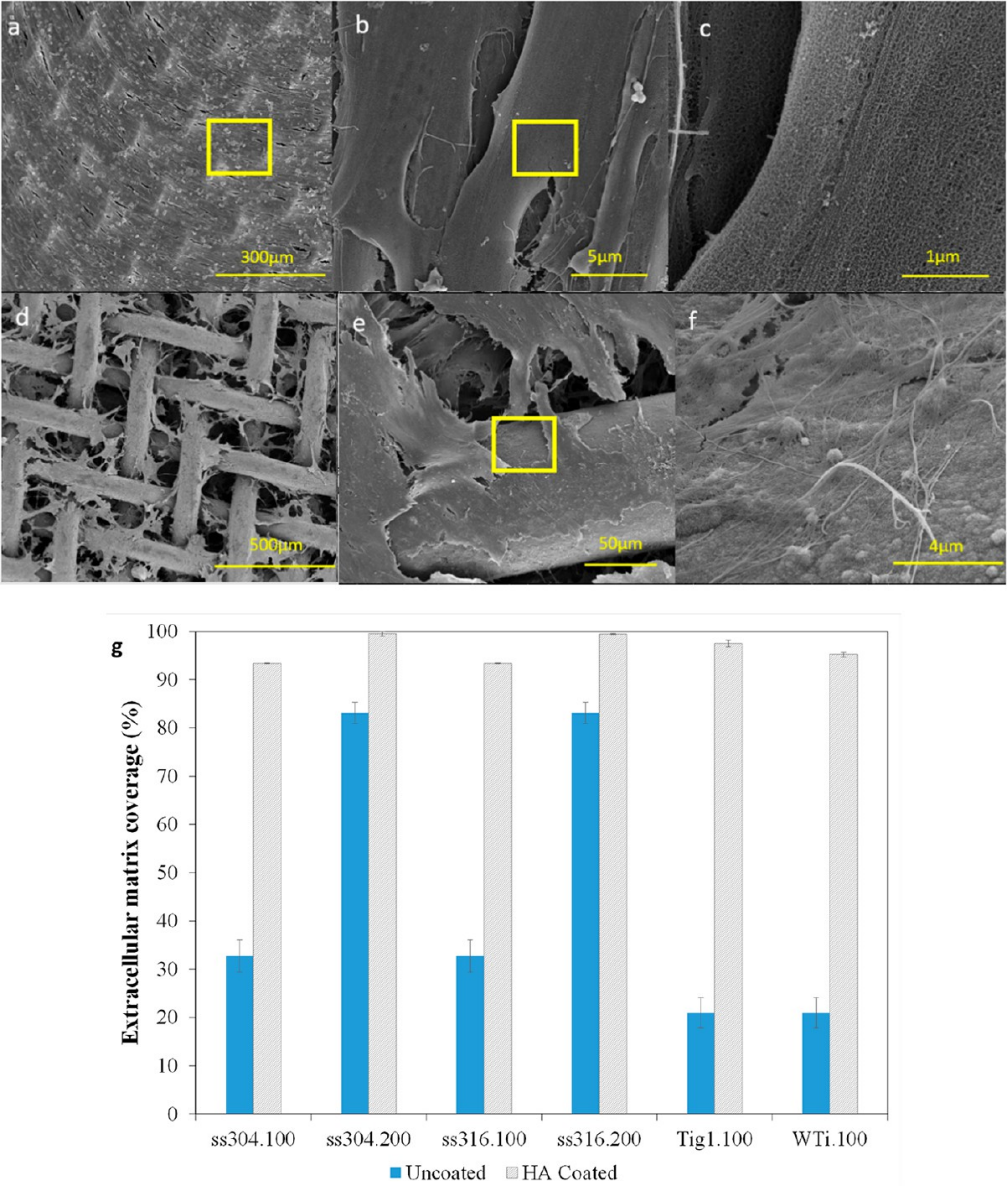
Representative FE-SEM images of extracellular matrix (ECM) on (a) HA-coated mesh ss304.200 and (b, c) close ups from squared areas, (d) partial ECM coverage on bare mesh Tig1.b, (e) junction of two wires in Tig1.b, and (f) cells and apatite particle on a wire, and (g) average % coverage by ECM (*n* = 3). Scale bar is 300 μm in panel a, 5 μm in panel b, 1 μm in panel c, 500 μm in panel d, 50 μm in panel e, and 4 μm in panel f. All comparisons between uncoated and HA-coated pairs are significant at ****p < 0.0001 (*n* = 3). All comparisons among HA-coated samples are not significant (ns, *n* = 3).

After 35 days, cells were collected from the substrates’ surface and counted using a hemocytometer (Figure 9). The values for the first control group (bare substrates in stromal media) are shown in the secondary vertical axis on the right. It can be concluded that ASCs could not survive or proliferate for 35 days in stromal media on uncoated metal mesh substrates. Although they can stay alive and differentiate on metal mesh substrates in osteogenic media, they only exhibited partial ECM coverage after 21 days, as will be discussed in section 3.4.2 and Figure 10. HA coating increased both proliferation in stromal medium and differentiation of ASCs on metal/ceramic mesh composite substrates in osteogenic medium (Figure 10).

#### 3.4.2 Cell Type and Tissue Surface Morphology

**Figure 11:**
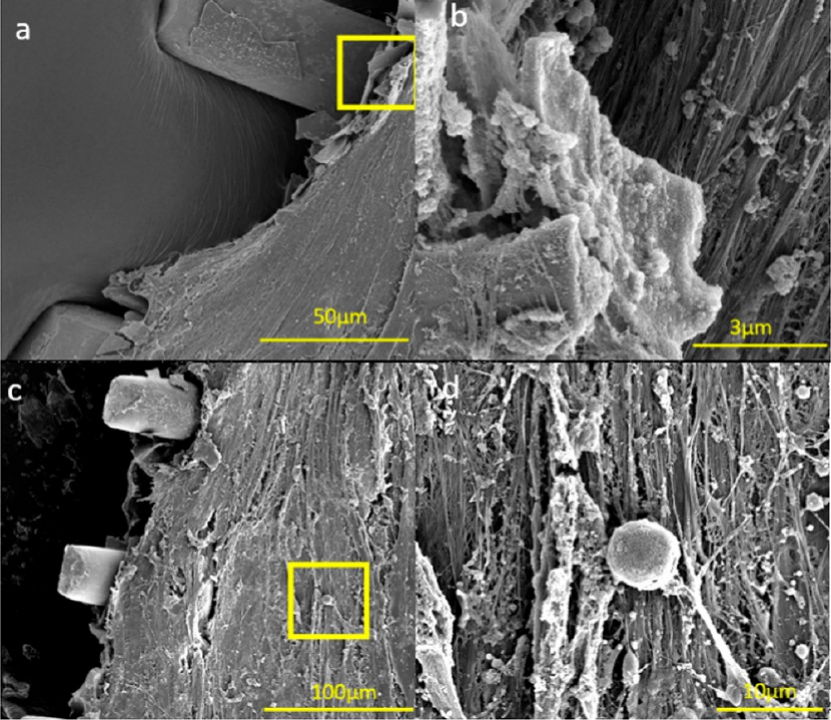
FE-SEM images for ECM covered mesh on both sides (a and c) and close ups of HA laminate (from squared areas) with apatite (b) and collagen fibers (d). Scale bar is 50 μm in panel a, 3 μm in panel b, 100 μm in panel c, and 10 μm in panel d.

Figures 10a–f and 11 show FE-SEM micrographs of ECM resulting from cell proliferation and differentiation on representative HA-coated and uncoated samples (for high and low ECM coverage). Both sides of each sample were examined under FE-SEM to confirm the 3D nature of the ECM network. Average ECM coverage % values are reported in Figure 10g. Overall, ECM coverage was more uniform on ss304 and ss316 HA-coated samples with mesh size 200 (>99.4%, Figure 10a–c and Figure 10g) compared to uncoated ones (<83.1%, Figure 10g). ECM coverage on HA-coated ss304, ss316, titanium grade 1, and white titanium with mesh size 100 was above 93%. In those cases, the wire peaks were not covered by ECM. The ECM coverage for uncoated samples with mesh size 100 was less than 34% (Figure 10d, e, and g). This difference shows the effect of mesh size on cell adhesion, proliferation, and ECM coverage through the mesh thickness. Collagen fibers, attached cells (Figure 10f and Figure 11d), and porous structure of multilayered ECM (Figure 10b and c) were found alongside the trapped HA laminates (Figure 11a and b).^45−47^ The microstructure of ECM on both uncoated and coated samples was similar. In addition, apatite formed on HA films and laminates as shown in Figures 10f and 11b.

Quality and coverage of ECM on HA-coated and uncoated substrates after 21 days revealed that ASCs can grow faster on substrates with HA coating for which the mesh holes remained open (as seen for % open mesh area with one or two HA layers in Figure 6j); cells can move from side to side and have a larger surface area to attach to and grow from. This resulted into a 3D ECM network. During imaging, it was noted that all samples were coated with nonconductive extracellular matrix. Therefore, from a design standpoint, HA-coated meshes with finer sizes and open areas above 15% would be preferable to create high ECM coverage in a 3D network.

Prior to immunocytochemistry analysis and TEM imaging, it was verified that ASCs could differentiate to bone cells by placing them into osteogenic media.^48^ To observe differentiation in ASCs, antibody staining for RUNX2 (osteoblasts at beginning of culture) and OPN (osteoblasts at the end of culture) was used to confirm cellular features using DAPI (nucleus) and phalloidin (cytoskeleton).

**Figure 12:**
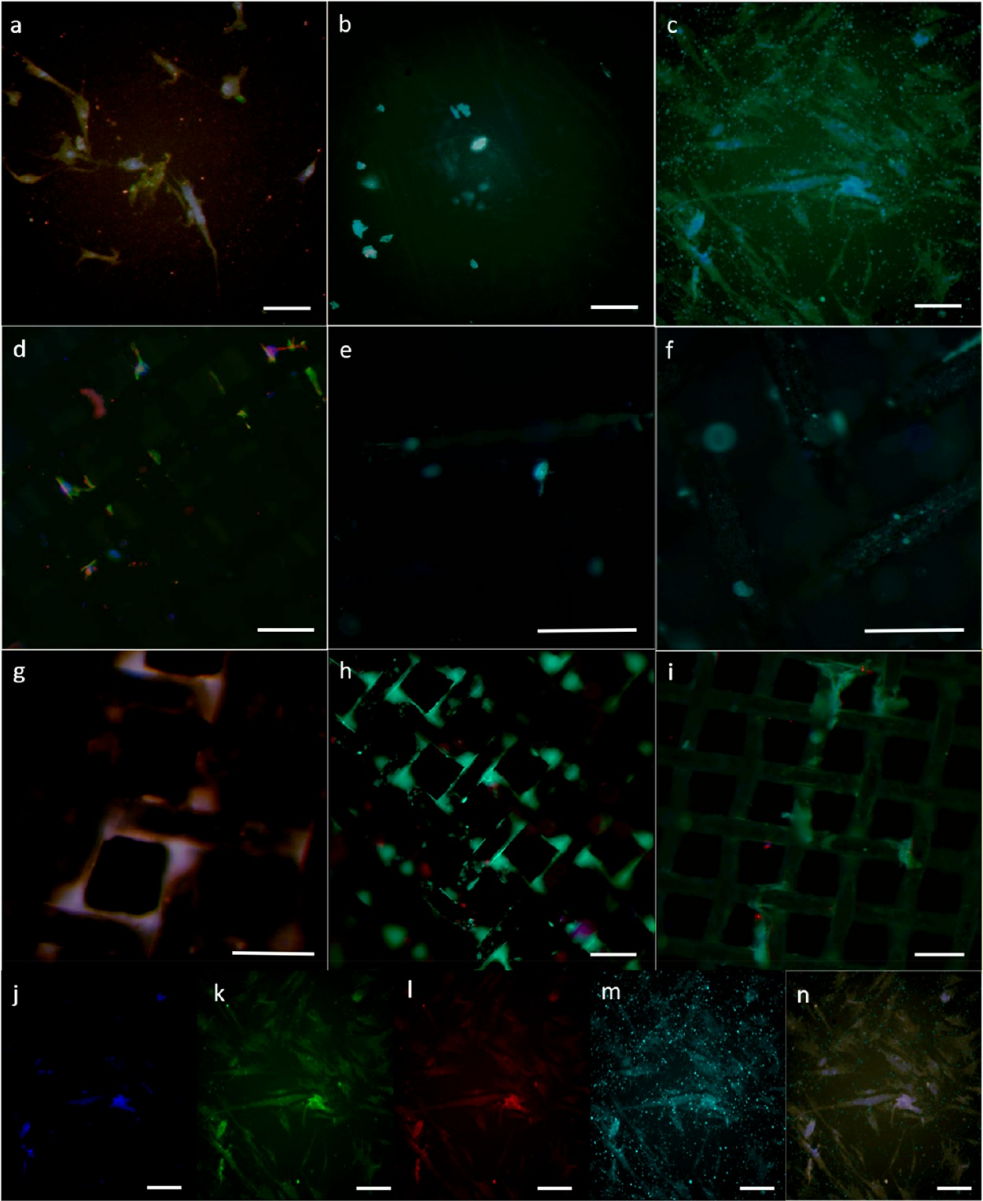
Fluorescence microscopy merged images showing nucleus, cytoskeleton, RUNX2, and osteopontin genes during osteogenic differentiation for three series of samples: blank well plate containing only ASCs for day (a) 7, (b) 14, (c) 21; bare titanium grade 1 (Tig1.b) (lowest ECM coverage) for day (d) 7, (e) 14, (f) 21; stainless steel 316. 200 (ss316.200.GS1) (highest ECM coverage) for day (g) 7, (h) 14, (i) 21. Fluorescent dyes represented for (j) nucleus, (k) cytoskeleton, (l) RUNX2, (m) osteopontin, and (n) merged image from j to m. Scale bar is 200 μm for all images.

Figure 12a–i shows merged images for immunocytochemistry results for cells attached on the bottom of the well plate (blank well plate contained only ASCs) and on the mesh substrates for those with the lowest and highest ECM coverage (as observed in Figure 10g for ss316.200 and Tig1.100). As the fluorescence images were directly acquired on the mesh substrates, some surface distortion was observed and all cells could not be captured in a single picture because of out-of-focus areas. Figure 12a–c were provided as a reference for blank well plate containing ASCs only. Figure 12j–n show the individual fluorescent dyes representing each part of the cells and the merged image that overlays all dyes. Fluorescent images captured for immunocytochemistry analysis showed high expression of gene RUNX2 at day 7, lower at day 14, and none at day 21 for all samples (Figure 12d–f for Tig1.b and Figure 12g–i for ss316.200.GS1). In reverse, OPN gene showed no presence during the first days, followed by an increased expression for day 14 and 21 for all samples. The coherency between transition from red (RUNX2) to blue (OPN) for all samples (blank well plate, Tig1.b, and ss316.200.GS1) shows there is no difference in osteogenic differentiation. Thus, bare metal or HA-coated metal meshes will not affect the quality of differentiation, while HA-coated samples can have higher ECM coverage and cell attachment (Figure 10). The bright points in Figure 12d–i indicate the position of cells on the mesh scaffold, which is mostly at the corners of the mesh openings.

**Figure 13:**
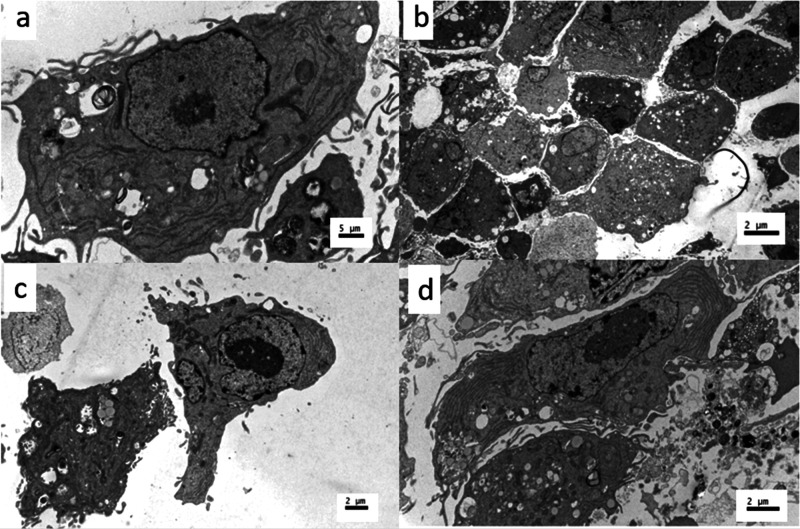
TEM images of bone cells collected from HA-coated samples: (a) osteogenic cell, (b and c) osteoblasts, and (d) deformed osteoblast. Scale bar is 5 μm in panel a and 2 μm in all others.

TEM images (Figure 13) confirmed different bone cells on uncoated and HA-coated samples (second control group and test group). The diversity of cells on coated samples was higher than uncoated ones as osteogenic cells and osteoblasts were found and are represented in Figure 13a–d. In Figure 13d, one osteoblast is differentiating to osteocyte, but the process is not complete yet. Again, it reveals HA coating can improve cells’ differentiation on metal/ceramic composite substrates. For uncoated samples, most of the cells were osteogenic cells.

Cell culturing and biocompatibility results showed that ECM could form in 21 days in contact with the appropriate medium on both sides of the HA-coated samples. Consequently, semiconductive metal/ceramic samples became nonconductive, covered at >99% by ECM containing real bone cells (stainless steel 304 and 316 HA-coated samples with mesh size 200). It confirms the potential for those samples to be used as cranioplasty implants, as they would be similar to bone tissue in the body after 21 days. The implant would also be able to keep cells alive with a suitable surface for cell proliferation in stromal media and cell differentiation in osteogenic medium.

### 3.5 Electrochemical Behavior Analysis

#### 3.5.1 Potentiodynamic Polarization

**Figure 14:**
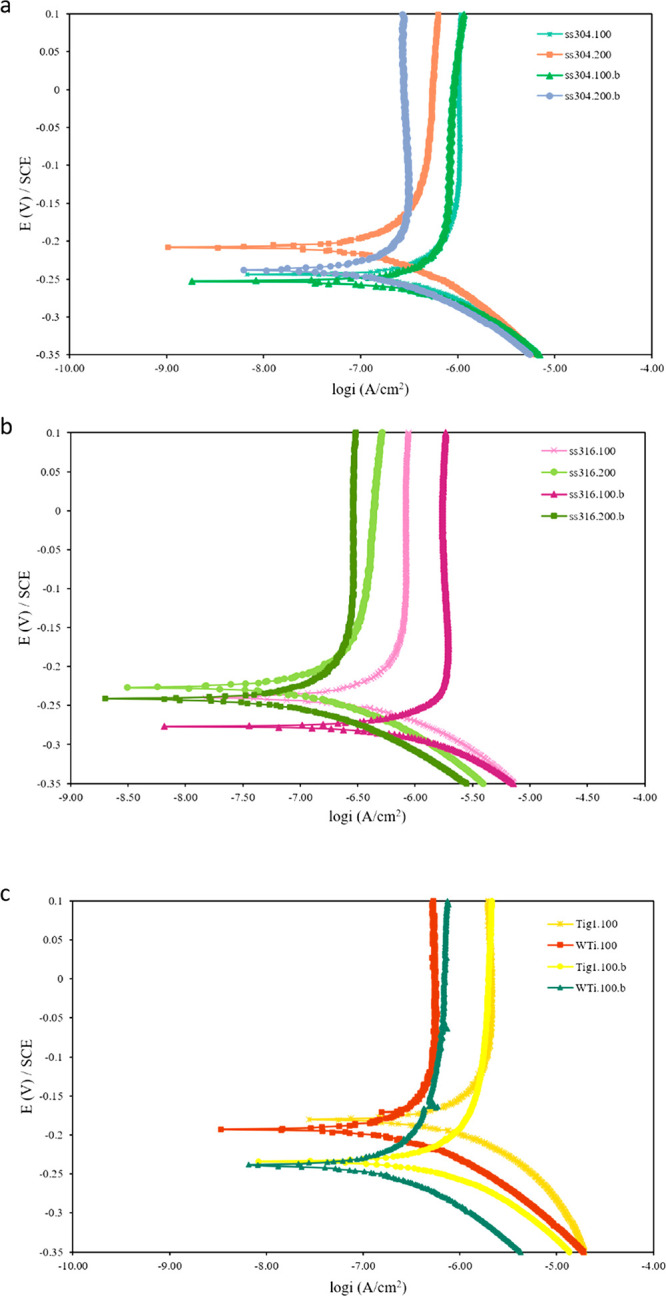
Potentiodynamic polarization curves for (a) ss304, (b) ss316, and (c) titanium, bare, and HA-coated samples, in simulated body fluid (SBF) at room temperature. Coating conditions for HA-coated samples were the same as those chosen for biocompatibility tests.

Potentiodynamic polarization trends were analyzed to determine the corrosion potential and current density for coated and uncoated samples for each material, in contact with SBF at room temperature (Figure 14). The coated samples were similar to the ones used for cell culture in osteogenic medium. Table 4 represents data for potentiodynamic polarization tests. Coated samples had higher *E*_corr_ (corrosion potential) than the uncoated one for all materials, which shows better corrosion protection performance for HA-coated samples than the uncoated substrates. For most coated samples, *E*_corr_ was higher than the highest *E*_corr_ value for uncoated samples, −0.234 V for titanium grade 1 (Tig1). It indicates HA coating in any condition can improve corrosion protection for both stainless steel and titanium, making the corrosion resistance ability of HA-coated stainless steel similar to that of bare titanium grade 1.

**Table 4:** Fitting Values for *E*_corr_ and *i*_corr_ in Potentiodynamic Polarization Curves and EIS Data^a^

	polarization data			EIS data				
sample code	*E*_corr_ (V) vs SCE	*i*_corr_ (μA/cm^2^)	*i*_passive_ (μA/cm^2^)	*R*_s_ (Ω·cm^2^)	*C*_dl_ (F/cm^2^)	*R*_ct_ (Ω·cm^2^)	*C*_C_ (F/cm^2^)	*R*_P_ (Ω·cm^2^)
ss304.100	–0.244 ± 0.002	0.49 ± 0.11	1.07	13.21	15.00 × 10^–5^	548	12.00 × 10^–5^	1.67 × 10^5^
ss304.100.b	–0.253 ± 0.006	0.46 ± 0.06	0.85	15.42	7.72 × 10^–5^	594	68.74 × 10^–5^	2.20 × 10^5^
ss304.200	–0.208 ± 0.007	0.18 ± 0.10	0.53	15.51	9.57 × 10^–5^	1011	9.67 × 10^–5^	3.73 × 10^5^
ss304.200.b	–0.238 ± 0.005	0.16 ± 0.04	0.29	14.55	2.89 × 10^–5^	698	2.74 × 10^–5^	5.97 × 10^5^
ss316.100.b	–0.277 ± 0.005	1.00 ± 0.15	1.75	14.83	5.46 × 10^–5^	831	9.36 × 10^–5^	1.17 × 10^5^
ss316.100	–0.240 ± 0.009	0.37 ± 0.13	0.83	13.28	7.92 × 10^–5^	795	5.96 × 10^–5^	2.65 × 10^5^
ss316.200	–0.227 ± 0.007	0.13 ± 0.05	0.42	18.27	6.35 × 10^–5^	1197	5.85 × 10^–5^	3.01 × 10^5^
ss316.200.b	–0.241 ± 0.004	0.11 ± 0.03	0.29	19.07	2.77 × 10^–5^	6321	5.03 × 10^–5^	3.11 × 10^5^
Tig1.100	–0.180 ± 0.003	0.74 ± 0.15	1.99	16.01	133.0 × 10^–5^	119	74.00 × 10^–5^	0.63 × 10^5^
Tig1.100.b	–0.234 ± 0.005	0.47 ± 0.11	1.95	19.41	2.77 × 10^–5^	609	34.00 × 10^–5^	0.24 × 10^5^
WTi.100	–0.193 ± 0.004	0.20 ± 0.06	0.57	16.76	22.70 × 10^–5^	113	8.37 × 10^–5^	1.80 × 10^5^
WTi.100.b	–0.238 ± 0.004	0.16 ± 0.04	0.69	17.78	2.78 × 10^–5^	1030	70.50 × 10^–5^	0.66 × 10^5^

a Samples for each material type are ordered according to *i*_corr_ values, from highest to lowest.

Corrosion current density (*i*_corr_) determines corrosion rate of metallic substrates, which has a direct relationship to material’s mass loss per year (Table 4). *I*_corr_ values for coated substrates were higher than bare substrates, which is not desirable. This is explained by the nonuniform and partial coating coverage on the surface, thus uncoated areas still exist. Galvanic corrosion occurred as HA-coated areas and bare metal are coupled. Consequently, *i*_corr_ for galvanic corrosion would be higher than *i*_corr_ for each couple. The lowest *i*_corr_ values were 0.11 and 0.13 μA/cm^2^ for uncoated and coated ss316.200, respectively. Ss316 is molybdenum-bearing grade and has better overall corrosion resistance than ss304. The mesh size 200 in both ss304 and ss316 possessed lower *i*_corr_ and higher *E*_corr_ than mesh size 100 because of its surface topography. The mesh diameter is smaller in mesh size 200 (*d* = 0.04 mm), thus wire bending is less significant at the wire junctions. Therefore, the surface topography is more uniform and flatter, and the area with localized corrosion on the surface is smaller. In general, the lowest *i*_corr_ was close to the range of HA coatings with good coverage found in the literature (0.007–0.1 μA/cm^2^)^49^ and better than most HA films on titanium alloy and ss316 L substrates (0.07–10 μA/cm^2^).^23,31^

**Figure 15:**
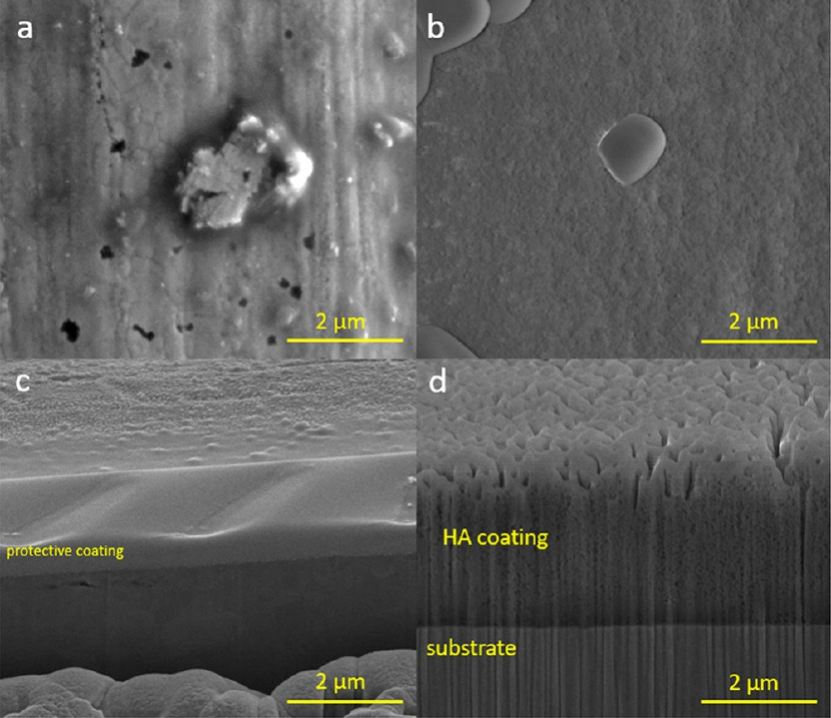
FE-SEM (SE) images for an example of corroded sample (ss304.200.CS1) after polarization in SBF, pits on passive layer (a), HA coating surface (b), and cross sections of uncoated area (c) and HA-coated area (d). Scale bar is 2 μm for all images.

In addition, Figure 14 shows that passivation occurred for all samples. *I*_passive_ was generally lower for ss316, as a passive layer can be formed at lower current and have higher corrosion protection. No pitting was observed for titanium samples in the range of −0.6 to 0.6 V (Figure 14c), but pitting and transpassive regions were observed on HA-coated ss304 (Figure 14a) and ss316 (Figure 14b) at about 0.35 V. Considering the galvanic corrosion case in which the bare substrate is the anode, passivation and pitting should happen for uncoated substrates. This means each sample has an HA film and a passive film of the metal oxide forming on its surface in contact with SBF. The results showed HA coating with semicoverage could be acceptable for titanium substrates, as bare substrates were covered by an oxide layer without any pitting. However, this is not desirable for ss304 and ss316 as the passive layer on bare metal pits. FE-SEM surface and cross-sectional images were taken to see the effect of the corrosive medium on bare and HA-coated samples. Figure 15a and b shows the surface of ss304.200.CS1 after polarization tests. Pitting occurred on uncoated areas of the surface, which had a passive layer (Figure 15a). The corresponding cross-section (Figure 15c) reveals that the passive layer is very thin and cannot be differentiated. There is good adhesion between HA layer and substrate as there is no delamination or formation of any other layer at the coating–substrate interface because of corrosion (Figure 15d).

#### 3.5.2 Electrochemical Impedance Spectroscopy

**Figure 16:**
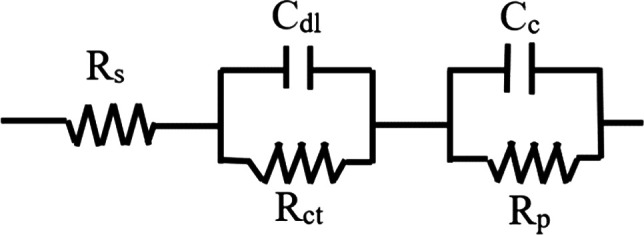
Equivalent electrical circuit for HA-coated samples used for EIS study.

**Figure 17:**
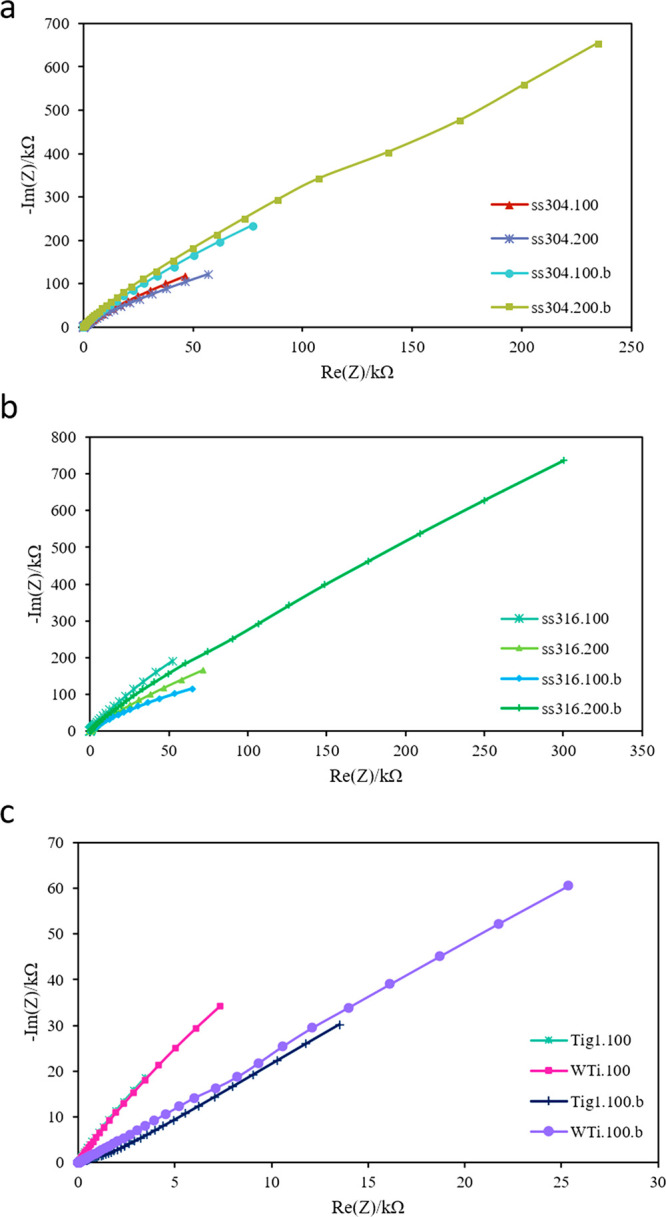
Nyquist plots of EIS data for (a) ss304, (b) ss316, and (c) titanium, bare, and HA-coated samples in simulated body fluid (SBF) at room temperature. Coating conditions for HA-coated samples were the same as those chosen for biocompatibility tests.

Electrochemical impedance spectroscopy was used to estimate the corrosion resistance of HA-coated samples. As the coating was not uniform, a related equivalent electrical circuit model for best fit was designed, as represented in Figure 16.^32,49^*R*_s_ is the solution resistance, or the SBF resistance, *C*_dl_ is the double layer capacitance, and *R*_ct_ is the charge transfer resistance. *C*_c_ is the interfacial capacitance, and *R*_p_ is the polarization resistance, inversely proportional to *i*_corr_ (from Table 4) and expressed as *R*_p_ = *B*/*i*_corr_, where *B* depends on material (substrate and coating) and solution.^32^ Here, *i*_passive_ is assumed equal to *i*_corr_, as the EIS test was done after polarization, and there were both HA and oxide layers on the wire surface. Four different materials were used in this study, and the trend of change in *R*_p_, according to *i*_passive_, should be considered for each material separately. Values of the circuit elements were calculated with ZSimpWin 3.20 from the Nyquist plots seen in Figure 17a–c, with parameters listed in Table 4, under the EIS data column. *R*_s_ is in the range of 13.0–19.5 Ω·cm^2^, showing solution consistency in polarization and EIS tests. *C*_c_ is on the order of 10^–4^–10^–5^, and *R*_p_ is on the order of 10^4^–10^5^. Lower *C*_c_ values represent lower transferring current, which means higher resistance (*R*_p_). *C*_c_, *R*_p_, and *i*_passive_ follow the same trend for each material and are on the same order of magnitude as reported in previous studies in the literature for HA-coated titanium and stainless steel.^23,32,49^

**Figure 18:**
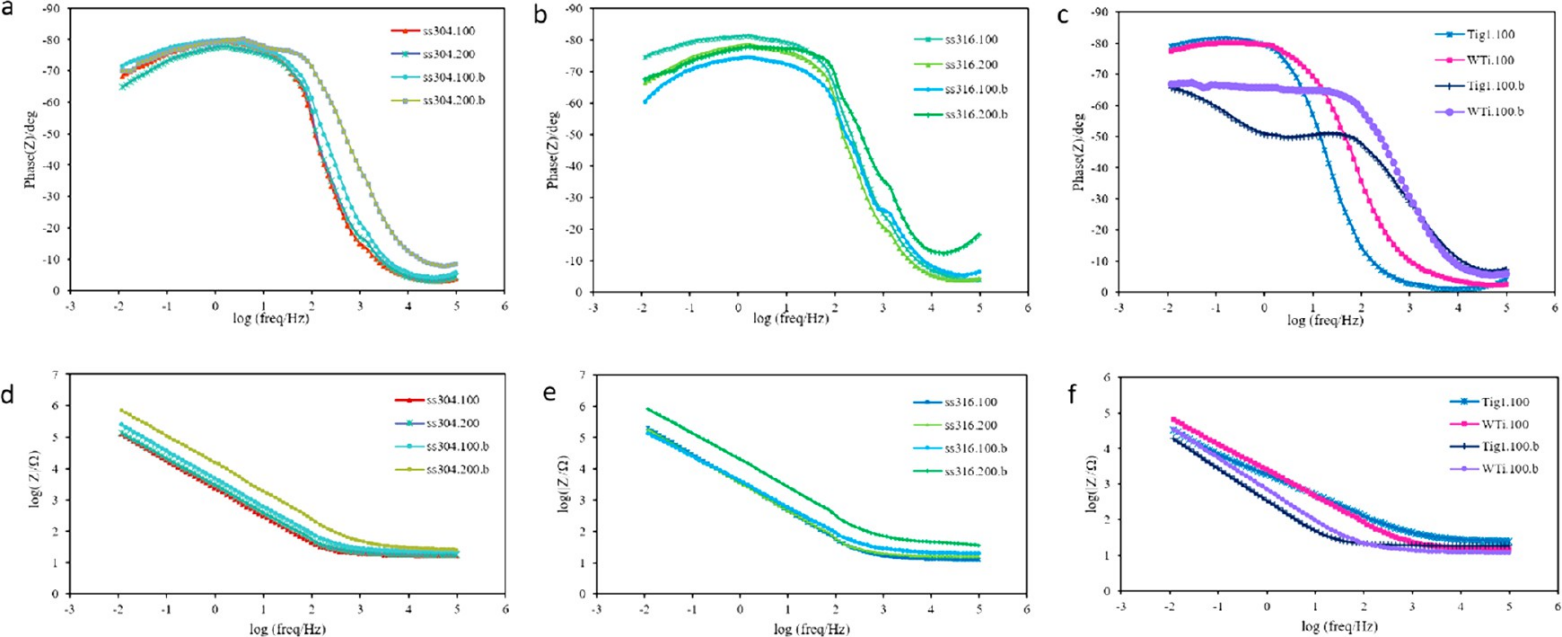
Bode phase plots (a) ss304, (b) ss316, and (c) titanium and bode impedance plots (d) ss304, (e) ss316, and (f) titanium of EIS data for bare and HA-coated samples, in simulated body fluid (SBF) at room temperature. Coating conditions for HA-coated samples were the same as those chosen for biocompatibility tests.

Bode phase angle and impedance plots are shown in Figure 18. In bode phase angle plots (Figure 18a–c), higher values at −90° indicate higher corrosion resistance, as −90° shows pure capacitance.^25^ For ss304, the uncoated samples, which were covered by an oxide layer, were higher around −81°, while HA-coated titanium samples displayed higher values around −81°. The results showed the same trends in the bode impedance plots (Figure 18d–f), in which corrosion resistance was better for stainless steel uncoated samples and titanium HA-coated samples.

Higher impedance in bode impedance plots indicates better corrosion resistance. Higher impedance in the low-frequency region represents resistance to mass transportation, while it shows propagation of charge transfer in the high-frequency region.^50^ For both ss304 and ss316, bode impedance plots for uncoated substrates reported higher values than coated ones because there is one uniform oxide layer covering the entire surface. Although pitting affected the oxide layer, the consequence was not visible in the plots. For titanium samples, the HA-coated ones led to higher values in the bode phase plot, showing higher resistance for coated samples, which is desirable.

## 4 Conclusion

In this study, we proposed the design of flexible, biocompatible composite implants by using a metal mesh as substrate and hydroxyapatite coating as bone regenerative stimulant derived from a simple sol–gel method. Experiments were carried out to understand the effect of coating method (dip-coating and drop casting), substrate material (stainless steel and titanium) and substrate mesh characteristics on implant’s performance.

Pure or biphasic nanorod hydroxyapatite coating on flexible mesh substrates were obtained through sol–gel method. All HA-coated samples dried at 150 °C in an oven possessed a crystalline structure. Different coating procedures and numbers of layers did not affect the crystal structure, shape or most intense plane reflections of the HA coating. It was observed that HA solutions with lower viscosity (GHA) led to higher wire and open areas coverage with the dip-coating process. Substrate material and wire diameter affected coating adhesion and coverage and consequently, coating thickness ranged between 4.0 to 7.9 μm for all samples. Smaller wire diameter (or higher mesh size) enhanced coating coverage and adhesion due to reduced wire bending at the junctions and smaller distance between wire peaks and valleys. Overall, adding more HA layers improved wire coverage (above 50%) and reduced open areas (less than 1%). However, application of more than one layer induced defects like microcracks and coating delamination. Hardness values for HA-coated stainless steel substrates were higher than titanium ones as adhesion of the HA coating was more uniform. Moduli of elasticity for most HA-coated samples were in the range of human skull’s modulus of elasticity (3.3–6.0 GPa), which is preferred for potential implants. Cell culture tests showed ASCs were more likely to attach and grow on samples that had open mesh areas after coating. Cell viability was higher than 90% after 3 days in stromal media and 21 days in osteogenic media. HA coating increased both proliferation and differentiation of ASCs on metal/ceramic mesh composite substrates. ECM developed into a 3D network on HA-coated samples for all mesh materials and its coverage area was between 93% and 99.5% (compared with 21% to 83% for bare substrates). Fluorescent imaging showed no antagonistic effect of the coatings on osteogenic differentiation. Finally, electrochemical behavior studies revealed that, even though corrosion protection for HA-coated samples was generally higher than bare samples, galvanic corrosion occurred on some samples. However, during use, a 3D ECM network covering the mesh implant could reduce the risk of galvanic corrosion.

Overall recommendations regarding design selection of mesh composite implants are summarized as follows: finest mesh size and dip-coating method to promote uniform coating on wires, and low number of HA layers to maintain open areas for 3D ECM formation. The experimental results indicated that, while HA-coated titanium grade 1 showed the best overall performance as a cranioplasty implant, HA-coated stainless steel 316 with mesh size 200 constitutes an adequate, lower cost alternative (by a factor above 100 based on raw materials cost). As potential of those mesh composites was demonstrated, future work would include in-depth analysis of in vivo response of the materials.

## Supporting Information Available

Image analysis for ECM coverage, timeline of biocompatibility experiments (for section 2.7), and summary of all samples conditions. (PDF)
