# Supplementary material for: Improved Biocompatible, Flexible Mesh Composites for Implant Applications via Hydroxyapatite Coating with Potential for 3-Dimensional Extracellular Matrix Network and Bone Regeneration
Source: ACS Appl Mater Interfaces. 2021 Jun 7;13(23):26824–40. doi: 10.1021/acsami.1c09034 (PMC8289173; doi:10.1021/acsami.1c09034)
Supplement: Supplementary file 1 — am1c09034_si_001.pdf [file am1c09034_si_001.pdf]

Supporting Information for:

Improved biocompatible, flexible mesh composites for implant applications via hydroxyapatite coating with potential for 3-dimensional extracellular matrix network and bone regeneration

Armaghan Naderi [a], Bin Zhang [a], Jorge A. Belgodere [b], Kaushik Sunder [a], Genevieve Palardy [a*]

[a] Department of Mechanical and Industrial Engineering, Louisiana State University, Baton Rouge, LA 70803, United States

[b] Department of Biological & Agricultural Engineering, Louisiana State University and Agricultural Center, Baton Rouge, LA 70803, United States

*Corresponding author: gpalardy@lsu.edu

In this file: Figures S1-S3

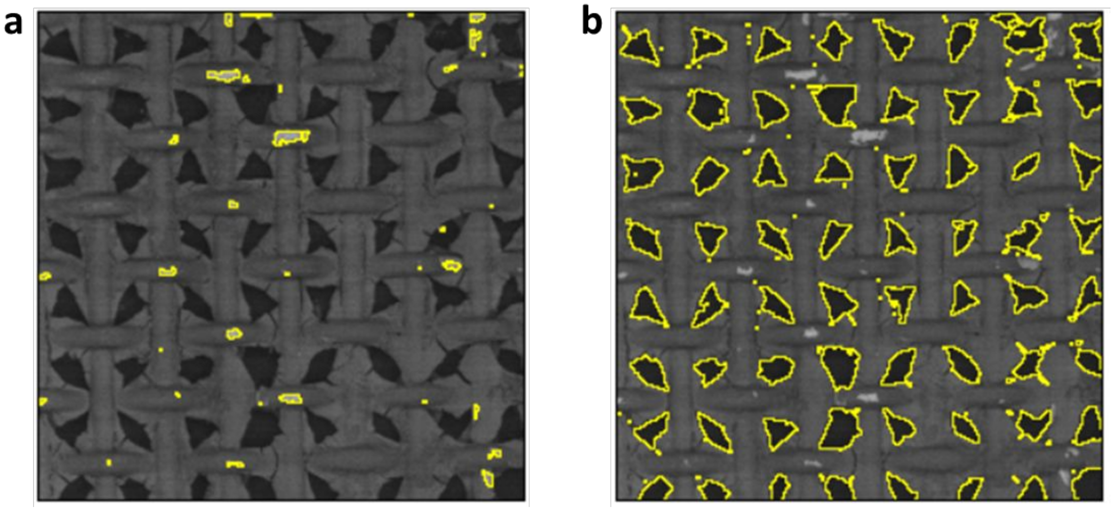

Figure S1. Example of image analysis to extract (a) uncoated wire area and (b) open mesh area (delineated in yellow). Mesh conditions: ss304 mesh size 200, coated by dip-coating with CHA solution and two HA layers.

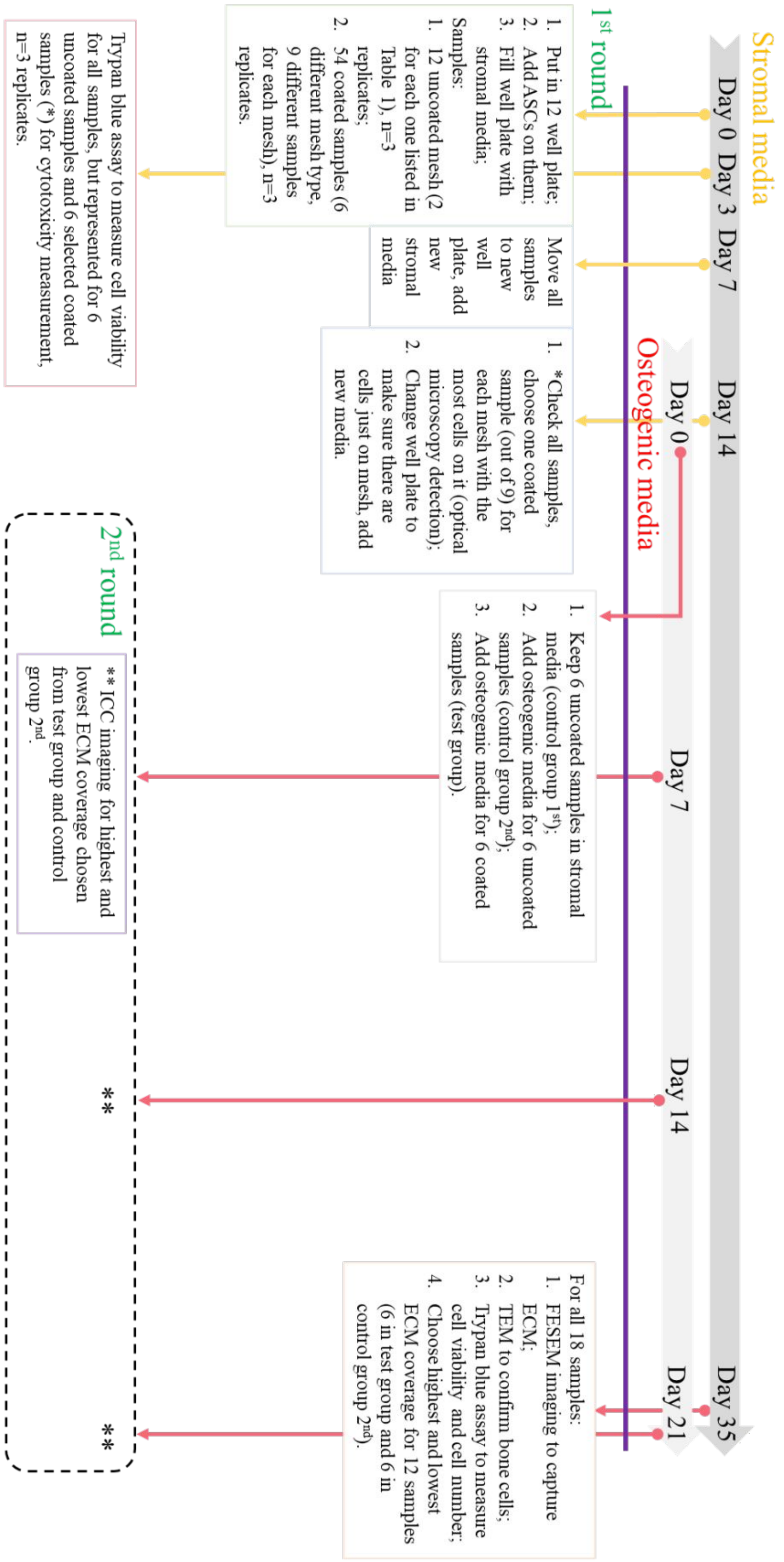

Figure S2. Timeline of biocompatibility experiments (Section 2.7 in manuscript).

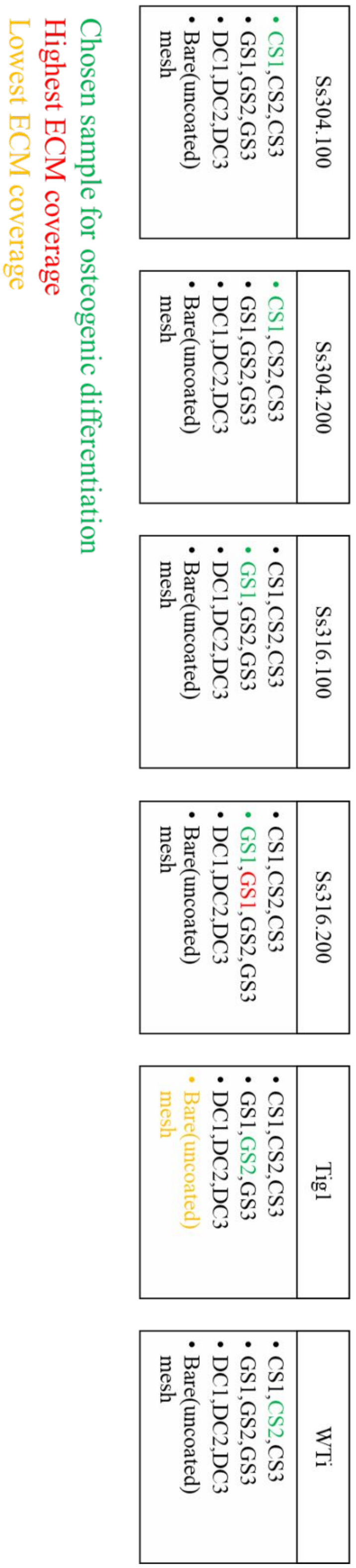

Figure S3. Summary of all mesh samples investigated in this study, along with those specifically selected for osteogenic differentiation (in green), and those with highest (in red) and lowest (in yellow) ECM coverage.
